# PLXDC2 siRNA‐Mediated Intervention Attenuates Microglial Senescence Through cGAS‐STING Signaling

**DOI:** 10.1002/advs.77793

**Published:** 2026-09-27

**Authors:** Heyue Lu, Zitian Zheng, Feiran Wang, Huanhuan Luo, Fei Ju, Yucheng Zhu, Zheng Zhou, Jie Sun, Zhenghui Qiu, Xiang Luo, Zhongzheng Jia, Jianquan Wang, Hongjie Huang, Bo Zhang

**Affiliations:** ^1^ Department of Radiology Affiliated Hospital and Medical School of Nantong University Nantong People's Republic of China; ^2^ Research Center of Clinical Medicine Affiliated Hospital and Medical School of Nantong University Nantong People's Republic of China; ^3^ Department of Sports Medicine Peking University Third Hospital Institute of Sports Medicine of Peking University Beijing People's Republic of China; ^4^ Beijing Key Laboratory of Research and Translation for Drugs and Medical Devices in Precision Diagnosis and Treatment of Sports Injuries Beijing People's Republic of China; ^5^ Engineering Research Center of Sports Trauma Treatment Technology and Devices Ministry of Education Beijing People's Republic of China; ^6^ Department of General Surgery Affiliated Hospital of Nantong University Nantong People's Republic of China; ^7^ School of Nursing Peking University Beijing People's Republic of China; ^8^ Department of General Surgery Affiliated Huaian No. 1 People's Hospital of Nanjing Medical University Huai'an People's Republic of China

**Keywords:** cellular senescence, cerium dioxide, ferritin, microglia, parkinson's disease

## Abstract

Parkinson's disease (PD) is driven by neurodegeneration, iron accumulation, and microglial senescence, yet effective therapy is hindered by the blood–brain barrier (BBB). By constructing the largest‐to‐date single‐cell atlas of the human substantia nigra, we identified a marked upregulation of PLXDC2 in PD microglia. Leveraging this finding, we developed a multifunctional biomimetic nanoplatform (HFn‐GM@siPLXDC2/DFO/CeO_2_‐NP). This system co‐encapsulates the iron chelator deferoxamine and antioxidant CeO_2_ nanoparticles, carries PLXDC2‐targeting siRNA, and features a ferritin‐modified microglial membrane coating to enhance BBB penetration and lesion targeting. In vivo, the nanoplatform efficiently traversed the BBB, reducing iron deposition and reactive oxygen species while suppressing microglial inflammation. Crucially, the treatment significantly alleviated dopaminergic neurodegeneration and improved motor performance‐including coordination, balance, and endurance in PD mice. Mechanistically, we demonstrate that PLXDC2 contributes to microglial senescence via the cGAS‐STING pathway, and its silencing attenuates neuroinflammation and oxidative stress. This study establishes a potent nanotherapeutic strategy integrating microenvironment remodeling with precise gene regulation to mitigate PD progression and alleviate motor deficits.

## Graphical abstract

PLXDC2 was identified as a microglial target in Parkinson's disease through human and mouse single‐cell analyses. Ferritin‐armed biomimetic nanovesicles delivered PLXDC2 siRNA, deferoxamine, and CeO_2_ nanoparticles across the blood–brain barrier, reducing cGAS‐STING activation, iron burden, oxidative stress, and senescence in microglia, thereby alleviating neuroinflammation, dopaminergic injury, and motor dysfunction in PD mice.

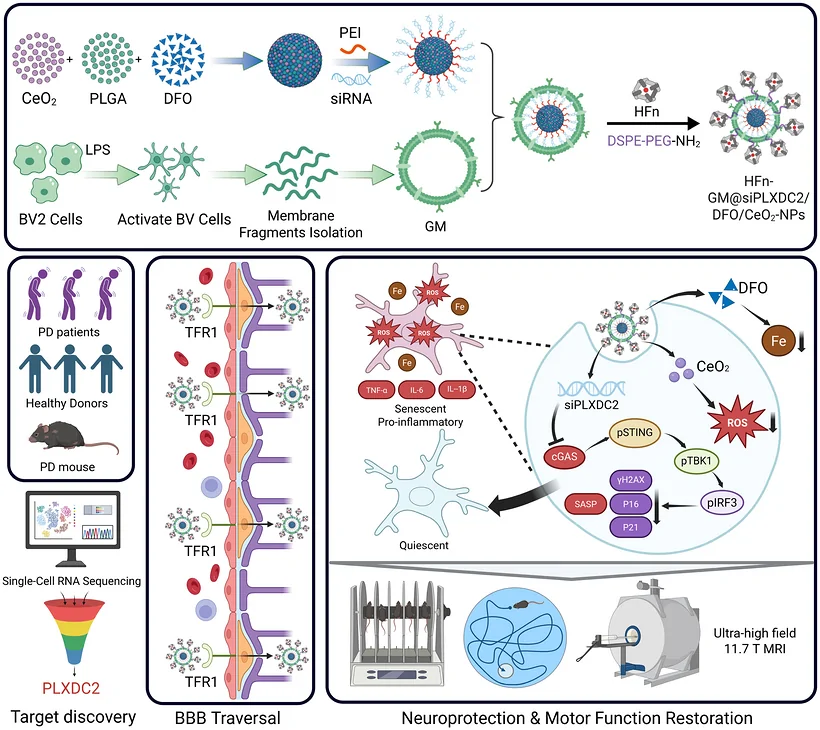

## 1 Introduction

Parkinson's disease (PD) is a common neurodegenerative disorder of the central nervous system (CNS), affecting more than six million individuals worldwide. Its principal pathological hallmarks include the progressive loss of dopaminergic neurons in the substantia nigra pars compacta and abnormal aggregation of α‐synuclein [1, 2]. Clinically, as the disease progresses, patients with PD gradually develop motor impairments such as resting tremor and bradykinesia, as well as a range of non‐motor symptoms, including cognitive decline. Current pharmacological treatments, which are primarily based on dopamine replacement therapy, can provide transient symptomatic relief without preventing disease progression [3, 4]. Accumulating evidence indicates that chronic neuroinflammation, dysregulated iron homeostasis, oxidative stress, and microglial dysfunction are closely implicated in PD pathogenesis, underscoring the urgent need to develop precision therapeutic strategies targeting the key pathological mechanisms of PD [5, 6].

In recent years, aberrant iron accumulation and excessive reactive oxygen species (ROS) generation in the substantia nigra have also been recognized as critical pathological features of PD. Iron overload exacerbates oxidative stress through Fenton chemistry, thereby promoting lipid peroxidation, mitochondrial dysfunction, and dopaminergic neuronal death. Multiple imaging studies and postmortem analyses have consistently demonstrated abnormal iron deposition within the substantia nigra of patients with PD, showing a positive association with disease severity [7, 8]. Additionally, neuroinflammation driven by microglial activation is a major contributor to PD onset and progression, as activated microglia release pro‐inflammatory cytokines, ROS, and nitric oxide, leading to neuronal injury [9]. Collectively, these findings suggest that the simultaneous modulation of iron metabolism, oxidative stress, and microglial dysfunction represents a rational and promising therapeutic strategy for PD.

Nanoparticle (NP) based drug delivery systems have attracted considerable attention in the neurodegenerative disease field owing to their tunable physicochemical properties, high drug loading capacity, and potential for multifunctional integration. However, poor blood–brain barrier (BBB) penetration, insufficient targeting specificity, as well as concerns regarding systemic toxicity and immunogenicity, have substantially limited their clinical application in PD [10]. To address these challenges, biomimetic strategies based on cell membrane coating have been progressively developed. Such approaches endow nanocarriers with the biological characteristics of natural cell membranes, thereby enhancing their in vivo stability, immune evasion capability, and targeted delivery efficiency [11, 12]. As the resident immune cells of the CNS, microglia are critically involved in PD pathogenesis and exhibit an intrinsic capacity to migrate toward inflamed and degenerating brain regions. Our previous studies have showed microglial membrane (GM) coated NP can significantly enhance accumulation within lesion sites in PD models and improve therapeutic efficacy [13]. Nevertheless, relying solely on microglial membrane coating remains insufficient to fully overcome the restrictive nature of the BBB, and further improvement in brain‐targeting efficiency is still urgently needed. Ferritin, a natural iron storage protein, has emerged as a promising brain targeting ligand due to its high affinity for transferrin receptor 1 (TFR1). TFR1 is reported to be highly expressed on brain microvascular endothelial cells in various neurodegenerative diseases, potentially as a compensatory response to iron homeostasis imbalance [14, 15]. These findings provide a theoretical basis for enhancing NP transcytosis across the BBB via ferritin mediated pathways. The heavy chain of ferritin (HFn), an endogenous iron binding protein, has been shown to facilitate BBB translocation through interactions with the iron transport system, conferring unique advantages for targeted drug delivery to the CNS [16, 17].

Based on the above background, we constructed a multifunctional biomimetic membrane based nanoplatform. First, the iron chelator deferoxamine (DFO) and ultrasmall cerium dioxide (CeO_2_) NP with potent antioxidant activity were co‐encapsulated into poly(lactic‐co‐glycolic acid) (PLGA) NP to synergistically counteract abnormal iron accumulation and ROS generation during PD pathogenesis. Subsequently, the NP were cloaked with GM to enhance their biocompatibility and targeting capability toward the neuroinflammatory microenvironment. On this basis, HFn was further incorporated onto the GM surface via hydrophobic insertion, thereby exploiting the upregulated iron transport pathways under PD conditions to improve BBB translocation and precise delivery to lesion sites. Accumulating evidence indicates that microglial dysfunction plays a pivotal role in the initiation and progression of PD. Through integrative analysis of single‐nucleus RNA (snRNA) sequencing data from PD patients and mouse models, we identified PLXDC2 as being markedly upregulated in PD microglia, suggesting its potential involvement in disease progression. Accordingly, in this study, siRNA targeting PLXDC2 was incorporated into the aforementioned biomimetic nanoplatform, aiming to downregulate PLXDC2 expression in microglia at the genetic level, thereby synergistically ameliorating the neuroinflammatory microenvironment and enhancing overall therapeutic efficacy. Functional studies demonstrated that this multifunctional biomimetic nanoplatform efficiently crossed the BBB in vivo and preferentially accumulated in the lesion‐affected regions of the brain in PD mouse models, significantly reducing local iron deposition and ROS levels, suppressing aberrant microglial activation and pro‐inflammatory cytokine release, and consequently alleviating dopaminergic neuronal injury and motor deficits. Mechanistically, PLXDC2 knockdown in microglia markedly attenuated cellular senescence phenotypes, decreased neuroinflammatory factor release and ROS production, and the cGAS‐STING signaling pathway was found to be essential for PLXDC2‐driven senescence‐associated responses.

## 2 Materials and Methods

### 2.1 Animal and Establishment of the PD Mouse Model

C57BL/6J mice (8 weeks old) were housed under specific pathogen‐free (SPF) conditions with ad libitum access to food and water. All procedures were approved by the Medical Ethics Committee of Nantong University (No. S20230228‐201). To induce Parkinson's disease (PD), mice received intraperitoneal injections of MPTP (30 mg/kg) daily for five consecutive days. This model was selected for its stable phenotypic consistency with PD dopaminergic loss. After model establishment, PD mice were randomly assigned to different treatment groups. After treatment, a series of behavioral tests were performed, including the Morris water maze test, open field test, traction test, pole test, and rotarod test. After completion of the behavioral assessments, mice were sacrificed at the therapeutic evaluation endpoint, and substantia nigra tissues were collected for TH immunohistochemistry, Nissl staining, Prussian blue staining, and measurements of ROS and iron levels. Figure S1 provides an overview of the animal experimental design to more clearly illustrate the overall in vivo experimental workflow.

### 2.2 Nuclei Isolation

Substantia nigra tissues were harvested from PD model and control mice, snap‐frozen, and stored in liquid nitrogen. Four samples per group were pooled for nuclei isolation. Thawed tissues were minced and lysed in pre‐chilled lysis buffer (10 mm Tris‐HCl, 10 mm NaCl, 3 mm MgCl_2_, 0.1% Nonidet P40). Lysates were filtered through 70 and 40 µm cell strainers to remove debris. After centrifugation (500 g, 10 min, 4°C), the nuclear pellet was resuspended in wash buffer (1 × PBS, 1% BSA, 0.2 U/µL RNase inhibitor), and the concentration was determined by cell counting.

### 2.3 Library Construction and Sequencing

Sorted nuclei were used for library construction using 10 × Chromium Next GEM Single Cell 3’ Reagent Kits and Gel Bead Kits v3.1. Library preparation was performed strictly according to the manufacturer's protocol, encompassing single‐nucleus encapsulation, cell lysis, mRNA capture, reverse transcription, and cDNA amplification. cDNA library quality was assessed using an Agilent 2100 Bioanalyzer system. Qualified libraries were sequenced on the Illumina NovaSeq platform.

### 2.4 Data Demultiplexing and Quality Control

Raw sequencing data (FASTQ format) were processed using Cell Ranger (10 × Genomics) for sample demultiplexing, alignment, and gene quantification. Quality control was performed in Seurat. Nuclei with 200–6000 detected genes were retained; cells with total UMI counts > 5000 were excluded to minimize doublets; and low‐quality cells with mitochondrial gene expression > 20% were removed. Genes expressed in fewer than three cells were filtered out.

### 2.5 Construction of a Single‐Cell Transcriptomic Atlas of the Substantia Nigra in PD Patients

To generate a comprehensive single‐cell transcriptomic atlas of the substantia nigra in PD patients, we integrated two publicly available snRNA sequencing datasets (GSE184950 and GSE243639) from the GEO database. Datasets were preprocessed in Seurat with consistent quality control (200–6000 genes, < 20% mitochondrial content). Expression matrices were normalized and batch‐corrected using the SCTransform method. Principal component analysis (PCA), UMAP dimensionality reduction, and unsupervised clustering were performed to generate a unified atlas for downstream annotation.

### 2.6 Cell–Cell Communication Analysis

PD‐associated remodeling of intercellular communication was analyzed using CellChat. Based on a ligand‐receptor interaction database, CellChat inferred signaling pathways and interactions among subpopulations. Global changes were assessed by comparing the number and strength of interactions between groups. Aggregated communication networks were constructed to identify major signaling directions. Differential pathways were ranked by Euclidean distances, and pathway activity changes were evaluated by quantifying information flow.

### 2.7 Preparation of PLGA/NP Co‐Loaded With Ultrasmall CeO_2_ NP and DFO

PLGA/NP co‐encapsulating ultrasmall CeO_2_ NP and DFO were prepared via a double emulsion (water‐in‐oil‐in‐water, W/O/W) solvent evaporation method. Briefly, DFO (20 mg) in deionized water formed the inner aqueous phase. Poly(lactic‐co‐glycolic acid) (PLGA) (100 mg) and CeO_2_ NP (5 mg) were dissolved in dichloromethane (4 mL) to form the oil phase. The phases were emulsified by probe sonication (80 W, 15 s), added to 200 mL of 2% (w/v) poly(vinyl alcohol) (PVA) aqueous solution, and sonicated again (60 W, 30 s). The emulsion was stirred overnight to evaporate the solvent. Unencapsulated DFO was quantified by high‐performance liquid chromatography (HPLC). NP were collected by centrifugation, washed, and characterized for size and zeta potential by dynamic light scattering (DLS) and morphology by transmission electron microscopy (TEM).

### 2.8 Preparation of siRNA‐Loaded PLGA/NP

PLGA/NP were surface‐modified with polyethyleneimine (PEI) to facilitate electrostatic binding of siRNA. The optimal complexation ratio was determined by mixing NP and siRNA at different nitrogen‐to‐phosphate (N:P) ratios (1:1 to 18:1). Binding efficiency was evaluated by agarose gel electrophoresis. For experiments, siRNA (2.5 nmol) was incubated with PEI‐modified PLGA/NP at room temperature for 20 min to form siRNA‐loaded PLGA/NP complexes.

### 2.9 Extraction of Microglial Cell Membranes

Microglial cells were washed with phosphate‐buffered saline (PBS), scraped, and pelleted by centrifugation. The pellet was resuspended in membrane protein extraction reagent A (containing 1% PMSF; Beyotime) and incubated on ice (4°C, 15 min). Cells were disrupted by ultrasonication and centrifuged (700 g, 10 min, 4°C) to remove nuclei. The supernatant was centrifuged (14 000 g, 30 min, 4°C) to isolate cell membrane fragments, which were stored at −80°C.

### 2.10 Preparation of HFn‐Modified Biomimetic Membrane‐Coated Drug‐Loaded NP

To prepare membrane‐coated NP, drug‐loaded cores were mixed with extracted cell membranes (1:2 protein:NP mass ratio) and co‐extruded through a 0.22 µm membrane. Separately, HFn was activated using EDC·HCl /NHS (5 mm/10 mm) in 0.1 m MES buffer (to activate the carboxyl groups on HFn into NHS esters) and conjugated to DSPE‐PEG‐NH_2_ (1:10 molar ratio) for 2 h. After quenching with glycine and purification via ultrafiltration, the DSPE‐PEG‐HFn conjugate was incubated with membrane‐coated NP (1:10 HFn:membrane protein mass ratio) at 37°C for 2 h to facilitate lipid insertion, yielding HFn‐modified biomimetic nanospheres.

### 2.11 Cell Culture

BV2 microglial cells (RRID:CVCL_0182), N2A neuroblastoma cells (RRID:CVCL_0470), and C8‐D1A astrocytes (RRID:CVCL_6379) were obtained from the Cell Bank of the Chinese Academy of Sciences. BV2 and C8‐D1A cells were cultured in Dulbecco's modified Eagle's medium (DMEM), and N2A cells in minimum essential medium (MEM). All media contained 10% fetal bovine serum (FBS) and 1% penicillin‐streptomycin. Cells were maintained at 37°C in 5% CO_2_. Moreover, to obtain the conditioned medium for microglia–neuron co‐culture, we collected the culture supernatants from BV2 cells under different treatment conditions and centrifuged them to remove cell debris. The resulting BV2‐conditioned medium was mixed with fresh complete N2A culture medium at a 1:1 ratio and then used to treat N2A cells to investigate the effect of PLXDC2 knockdown in microglia on neuronal cell viability.

### 2.12 In vitro BBB Model

An in vitro BBB model was established using a Transwell co‐culture system. Mouse brain microvascular endothelial cells (bEnd.3) were seeded in the upper chamber, while BV2 microglial cells were cultured in the lower chamber. After 48 h of co‐culture, a stable endothelial monolayer with tight junctions was formed. BBB permeability was evaluated using coumarin‐6 (C6) labeled NP (GM@NP^C6^ and HFn‐GM@NP^C6^). Under light‐protected conditions, NP were added to the upper chamber and incubated for predetermined time intervals. BV2 cells in the lower chamber were then collected, and intracellular fluorescence intensity was quantified by flow cytometry and visualized by fluorescence microscopy to assess NP translocation across the BBB.

### 2.13 Protein Extraction and Western Blotting

Samples were lysed in RIPA buffer with protease/phosphatase inhibitors and centrifuged. Protein concentrations were determined using the BCA assay. Proteins were separated by SDS‐PAGE and transferred to nitrocellulose membranes. Membranes were blocked with 5% nonfat milk and incubated overnight at 4°C with primary antibodies. After washing with TBST, membranes were incubated with HRP‐conjugated secondary antibodies for 1 h at room temperature. Signals were detected using enhanced chemiluminescence and quantified using an imaging system.

Primary antibodies used included PLXDC2 (#PA5‐20467, Thermofisher), TBK1 (3504T, Cell Signaling Technology), Phospho‐TBK1 (5483T, Cell Signaling Technology), cGAS (31659T, Cell Signaling Technology), STING (13647T, Cell Signaling Technology), Phospho‐STING (72971T, Cell Signaling Technology), IRF3 (4302T, Cell Signaling Technology), Phospho‐IRF3 (29047T, Cell Signaling Technology), p16 (23200T, Cell Signaling Technology), p21 (2947T, Cell Signaling Technology), H2AX (F0284, Selleck), Phospho‐H2AX (2577S, Cell Signaling Technology), GAPDH (2118T, Cell Signaling Technology), beta‐Actin (4970T, Cell Signaling Technology).

### 2.14 Histological Analysis

Brain tissues were fixed in 4% paraformaldehyde, embedded in paraffin, and sectioned (5 µm). Following antigen retrieval in EDTA buffer (pH 9.0) and blocking with 3% BSA, sections were incubated overnight at 4°C with primary antibodies against tyrosine hydroxylase (TH; #GB12181, Servicebio), IBA1 (#GB12105, Servicebio), and PLXDC2 (#PA5‐20467, Thermofisher). Signals were detected using HRP‐conjugated secondary antibodies (anti‐rabbit: #G1213, Servicebio; anti‐mouse: #G1214, Servicebio) with DAB or fluorophore‐conjugated secondary antibodies (#RGAR002, #RGAM002, #RGAR004, #RGAM004, Proteintech) with DAPI counterstaining. Images were acquired using a Leica microscope. Iron deposition was visualized using a Prussian blue staining kit (#G1428, Solarbio) per the manufacturer's instructions. For cellular immunofluorescence, cells were fixed (4% PFA), permeabilized (0.3% Triton X‐100), blocked (5% BSA), and incubated with primary antibodies followed by fluorophore‐conjugated secondary antibodies.

### 2.15 Senescence Associated β‐Galactosidase (SA‐β‐gal) Staining

Microglial senescence was assessed using a β‐galactosidase staining kit (#C0602, Beyotime). Fixed BV2 cells were incubated with the staining working solution overnight at 37°C in a non‐CO_2_ incubator. The percentage of SA‐β‐gal‐positive cells (blue) was quantified in three randomly selected fields to evaluate cellular senescence.

### 2.16 Nile Red Staining

Lipid droplet accumulation was quantified by Nile Red staining. Cells were incubated with a working solution (diluted 1:100 in PBS from a 10 µg/mL stock) for 30 min in the dark. Fluorescence intensity was subsequently measured by flow cytometry.

### 2.17 RNA Extraction and Quantitative Real‐Time PCR (qPCR)

Total RNA was extracted using TRIzol and reverse‐transcribed using the Script First Strand c‐DNA Synthesis SuperMix (Takara). qPCR was performed with SYBR Green Master Mix (Takara) on a QuantStudio 6 Flex system using GAPDH as the reference. Relative expression was calculated via the 2^−ΔΔCt method. Primer sequences were:

IL‐1β (F: 5’‐GCAACTGTTCCTGAACTCAACT‐3’,

R: 5’‐ATCTTTTGGGGTCCGTCAACT‐3’);

IL‐6 (F: 5’‐TAGTCCTTCCTACCCCAATTTCC‐3’,

R: 5’‐TTGGTCCTTAGCCACTCCTTC‐3’);

TNF‐α (F: 5’‐CAGGCGGTGCCTATGTCTC‐3’,

R: 5’‐CGATCACCCCGAAGTTCAGTAG‐3’);

PLXDC2 (F: 5’‐GCCGCAGCAGGAGTTATGTTA‐3’,

R: 5’‐TTCATTCCAAGGAAAAGCGTTTG‐3’).

### 2.18 Flow Cytometry

Substantia nigra tissues were dissociated using a Miltenyi mouse brain tissue dissociation kit. Single‐cell suspensions were filtered and stained with antibodies against NeuN (#CL488‐66836, Proteintech), GFAP (#53‐9892‐80, Invitrogen), or IBA1 (#CL488‐81728, Proteintech) for 30 min at 4°C. Data were acquired by flow cytometry and analyzed using FlowJo software. In addition, the intracellular ROS levels in BV2 cells were measured using a commercial ROS assay kit from Beyotime Biotechnology (#S0033S) according to the manufacturer's instructions. Briefly, BV2 cells from different treatment groups were incubated with the ROS detection probe, washed to remove excess probe, and then analyzed by flow cytometry.

### 2.19 ELISA Assay

The levels of IL‐1β, IL‐6, and TNF‐α in BV2 cell supernatant were detected using commercial ELISA kits from Elabscience according to the manufacturer's instructions. Briefly, the samples and standards were added to antibody‐coated plates and incubated with the corresponding biotinylated detection antibodies, followed by horseradish peroxidase‐conjugated avidin. After washing, TMB substrate was added for color development, and the reaction was stopped with stop solution. The absorbance was measured at 450 nm using a microplate reader, and cytokine concentrations were calculated based on the standard curves.

### 2.20 Mouse MRI and Quantitative Susceptibility Mapping (QSM) Reconstruction

The brain imaging procedures for establishing the PD model and subsequent QSM reconstruction were performed on an 11.7 T Bruker Biospec scanner (Bruker, Ettlingen, Germany). This system features a horizontal bore design and incorporates actively shielded gradient coils capable of producing pulsed field gradients up to 0.74 T/m. For signal transmission and reception, a 72‐mm quadrature volume coil and a four‐channel phased array coil (configured in a 2 × 2 arrangement) were employed, respectively. Whole‐brain phase data, intended for QSM processing, were obtained with a conventional three‐dimensional flow‐compensated spoiled gradient echo (SPGR) sequence. The acquisition parameters were set as follows: imaging matrix = 170 × 150 × 70, field of view = 17 × 15 × 7 mm^3^, flip angle = 20°, echo time (TE) = 2.216 ms, repetition time (TR) = 100 ms, and slice thickness = 7.0 mm. The total duration for this scan was 17 min and 30 s.

### 2.21 Morris Water Maze Test

The Morris water maze was used to assess spatial learning and memory. The apparatus consisted of a 120 cm circular pool (20°C ± 2°C) with a platform submerged 1 cm below the surface. During the 4‐day training phase (4 trials/day), mice were allowed 60 s to locate the hidden platform; those failing to find it were guided to the platform and allowed to stay for 10 s. On day 5, a probe trial was conducted with the platform removed. The time spent in the target quadrant and the number of platform crossings were recorded to assess memory retention.

### 2.22 Pole, Traction, Rotarod, and Open Field Test

Motor function and exploratory behavior were evaluated using a battery of behavioral tests. Pole test: Mice were placed head‐down on the top of a vertical rough‐surfaced pole (50 cm). The time taken for both forepaws to reach the base was recorded, and the mean descent time from three trials was calculated to assess motor coordination. Traction test: each mouse was placed on a horizontal wire grid and allowed to hang, and the latency to fall was recorded. Each mouse was tested three times, and the average latency was used for analysis. A maximum cutoff time of 60 s was applied. In addition, the total distance traveled and number of central area entries were analyzed using EthoVision XT 16 software. Rotarod test: Prior to testing, mice underwent an acclimation session (linear acceleration from 4 to 40 rpm over 5 min). Motor endurance was then evaluated at a constant speed of 40 rpm. The latency to fall was recorded as the average of three trials. Open field test: Mice were placed in the center of a white square arena (40 × 40 × 40.5 cm) and allowed to explore freely for 10 min. Locomotor trajectories were recorded via a video‐tracking system to analyze the total distance traveled and frequency of entries into the central zone.

### 2.23 Statistical Analysis

After MPTP modeling, mice that met the experimental inclusion criteria were randomly allocated to the indicated treatment groups before drug administration using a computer generated random number sequence. Behavioral testing, histological image acquisition and quantification, flow cytometric analysis, and statistical analysis of major endpoints were performed by investigators who were blinded to group allocation whenever feasible. Predefined exclusion criteria included death unrelated to the intended experimental intervention, failed administration or sample collection, severe technical artifacts that precluded reliable quantification, and inability to complete behavioral testing due to causes unrelated to the PD phenotype. Exclusions were determined before final statistical analysis. Processing and analysis of snRNA sequencing data were primarily performed using the R programming environment (version 4.5.1) together with relevant supporting packages. All other experimental data were statistically analyzed using GraphPad Prism 9 software, and results are presented as the mean ± standard error of the mean (SEM) unless otherwise stated. For comparisons between two groups, Student's *t*‐test was applied. When comparisons among three or more groups were required, one‐way analysis of variance (ANOVA) followed by Tukey's post hoc multiple‐comparison test was performed. All statistical tests were two‐sided, and *p* < 0.05 was considered statistically significant.

## 3 Results and Discussion

### 3.1 snRNA‐seq Reveals Cellular Heterogeneity and Compositional Complexity of the Substantia Nigra in PD

**FIGURE 1:**
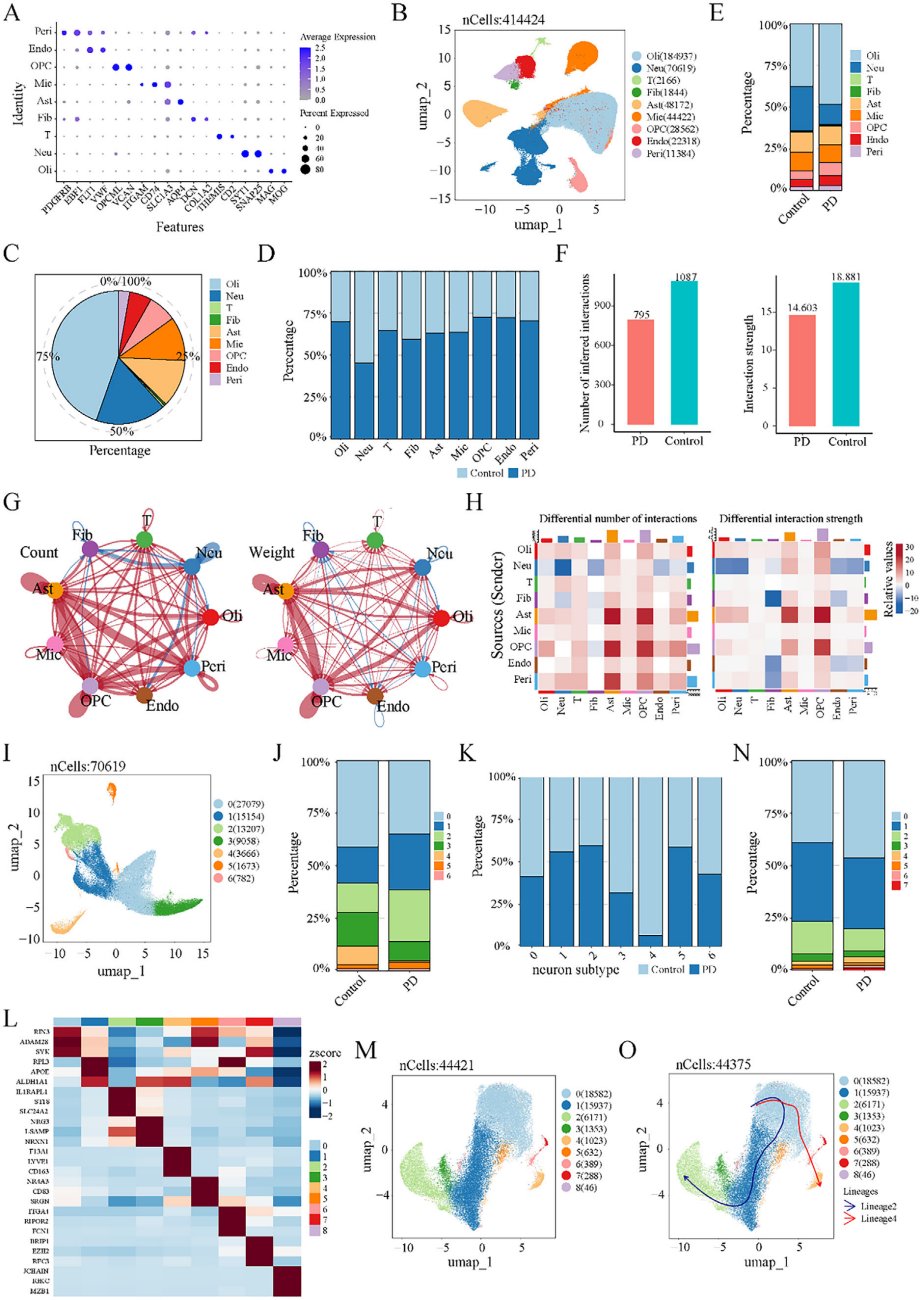
Single‐nucleus transcriptomic landscape of the substantia nigra in PD patients. (A) Expression levels of canonical cell‐type‐specific markers used to identify cellular subtypes in human substantia nigra tissue; (B) UMAP visualization of cell clustering. Oli, oligodendrocytes; Neu, neurons; T, T cells; Fib, fibroblast‐like cells; Ast, astrocytes; Mic, microglia; OPC, oligodendrocyte progenitor cells; End, endothelial cells; Peri, pericytes; (C) Pie chart showing the proportional composition of each cell population in the substantia nigra; (D) Distribution of cell proportions across different cell populations stratified by disease status; (E) Comparison of cell‐type proportions between the PD group and healthy controls; (F) Number (left) and strength (right) of intercellular interactions inferred by CellChat; (G) Network plots depicting the number and strength of CellChat‐inferred cell–cell communications; (H) Heatmap illustrating the number and strength of CellChat‐inferred cell–cell communications; (I) UMAP visualization of reclustered neuronal subpopulations; (J) Proportional distribution of neuronal subtypes in the PD group and healthy controls; (K) Distribution of neuronal subtype proportions stratified by disease status; (L) Expression levels of marker genes across microglial subpopulations; (M) UMAP visualization of reclustered microglial subpopulations; (N) Proportional distribution of microglial subtypes in the PD group and healthy controls; (O) Pseudotime trajectory analysis illustrating the inferred transcriptomic continuum of microglial states.

To investigate alterations in the proportions and functional states of distinct cellular subpopulations in the substantia nigra during PD initiation and progression, we collected snRNA‐seq data from postmortem substantia nigra tissues of 38 patients with PD and 23 healthy controls, thereby constructing the largest single‐cell atlas of PD substantia nigra to date. Using a Seurat‐based standardized data preprocessing and clustering pipeline‐including quality control, Harmony‐based batch correction, cluster stability assessment, and doublet detection [18, 19], we obtained 414 424 high‐quality nuclei (Figure S2A). Based on a set of cell‐type‐specific marker genes, nuclei were classified into major CNS cell types, including oligodendrocytes (MAG and MOG), microglia (CD74 and ITGAM), astrocytes (AQP4 and SLC1A3), neurons (SYT1 and SNAP25), oligodendrocyte precursor cells (OPCs; VCAN and OPCML), endothelial cells (FLT1 and VWF), T cells (THEMIS and CD2), and pericytes (PDGFRB and EBF1) (Figure 1A and Figure S2B–J) [20, 21, 22]. UMAP analysis of the integrated PD and control datasets, combined with cell‐type annotation, identified nine distinct cell types. The number of cells in each subgroup ranged from 184 937 (oligodendrocytes) to 2166 (T cells), and both PD and control samples contained all nine identified cell populations. (Figure 1B and Figure S3A,B). In the human substantia nigra, oligodendrocytes constituted the most abundant cell population, followed by neurons, microglia, astrocytes, OPCs, endothelial cells, pericytes, T cells, and fibroblasts (Figure 1C). Consistent with previous studies, calculation of relative cell proportions revealed a marked decrease in neurons and an increased proportion of microglia in PD samples compared with controls (Figure 1D,E). We further identified highly expressed genes in each cell population and performed GO enrichment analyses (Figure S4 and Table S1). Microglia were predominantly enriched in processes related to immune system regulation, leukocyte migration and activation, inflammatory responses, and cytokine secretion. Oligodendrocytes were mainly associated with myelination, ensheathment of neurons, and axon ensheathment, whereas neuronal subpopulations were enriched in vesicle‐mediated transport at synapses, neurotransmitter secretion, and synaptic signal release. These GO enrichment results strongly supported the accuracy of our cell‐type annotations (Figure S4). Functional enrichment analyses suggested that intercellular communication among distinct cell populations in the substantia nigra may be altered in PD. To further investigate changes in cell–cell interactions during PD progression, we employed CellChat to infer intercellular communication networks. Compared with healthy controls, PD samples exhibited a pronounced reduction in both the quantity and robustness of inferred intercellular communications (Figure 1F). In particular, microglia displayed substantially enhanced interaction frequency and interaction strength with multiple neighboring cell populations in the PD condition (Figure 1G,H). We next examined L‐R pairs and signaling pathways contributing to these alterations and found that CDH signaling activity was significantly increased in PD, whereas PDG, ADGRB, CNTN, and other pathways were downregulated (Figure S5A,B).

To further delineate neuronal cell types affected by PD, we re‐clustered neuronal nuclei and identified seven distinct subpopulations with unique gene expression signatures, designated Neuron 0 to Neuron 6 (Figure 1I). All neuronal subclusters were present in both PD and control samples (Figure S6A). However, compared with controls, the numbers of Neuron 0, Neuron 3, and Neuron 4 were significantly reduced in PD samples, whereas Neuron 1 and Neuron 2 were significantly increased (Figure 1J,K). To characterize these neuronal populations, pathway overrepresentation analyses were performed on the marker genes of each subcluster. Neuron 0 and Neuron 5 exhibited elevated levels of representative GABAergic genes, such as GAD1, GAD2, and GABRA1, thereby supporting their classification as inhibitory neuronal subtypes. These subpopulations also expressed genes involved in dopamine secretion, metabolism, and transport (SYT2 and CPLX1), suggesting highly efficient and tightly regulated synaptic output. Pathway analysis of marker genes demonstrated strong associations with synaptic transmission, GABAergic signaling, and synaptic vesicle exocytosis (Figure S6B,C and Table S2). Neuron 3 represented a second neuronal population that was markedly reduced in PD patients. This subpopulation was characterized by the expression of key dopaminergic neuron markers, including TH, SLC6A3, SNCA, and ALDH1A1, indicating that dopaminergic neurons constitute the primary neuronal subtype lost in PD (Figure S6B,C and Table S2).

Our subsequent investigation into microglial heterogeneity under PD conditions revealed nine distinct subclusters (Microglia 0–8), as shown in Figure 1M. Although all clusters were detected in both groups (Figure S7A), their proportions shifted significantly. Specifically, PD samples exhibited a marked expansion of Microglia 0 and 4, whereas Microglia 2 and 3 showed a substantial reduction (Figure 1N). Microglia 0, characterized by representative markers such as RIN3, ADAM28, and SYK, exhibited pronounced immune activation (Figure 1L). In addition, neuroinflammation‐associated genes such as DOCK8 and senescence‐related ARHGAP family transcripts were significantly upregulated in this subpopulation (Figure S7B and Table S4). Collectively, these findings suggest that Microglia 0 represents a pro‐inflammatory subtype that plays a critical role in PD pathogenesis and progression. By ordering microglial cells along pseudotime, we explored the transcriptomic continuum of microglial states under PD conditions and identified two pseudotime associated trajectory branches (Figure 1O). One branch was enriched for inflammatory‐state markers, including SIGLEC1, MARCO, CR1, VAV3, and DAPK1, and GO analysis showed enrichment of Fc receptor signaling, phagocytosis, and receptor‐mediated endocytosis (Figure S7C). Another branch was characterized by reduced representation of Microglia 0–3 in PD samples, with GO terms related to neuronal physiological functions and synaptic homeostasis. These findings collectively support that microglial alterations in PD are closely linked to both inflammatory responses and neuronal functional impairment (Figure S7C).

### 3.2 snRNA Sequencing Reveals Upregulation of PLXDC2 in Microglia from the Substantia Nigra of MPTP‐Treated Mice

**FIGURE 2:**
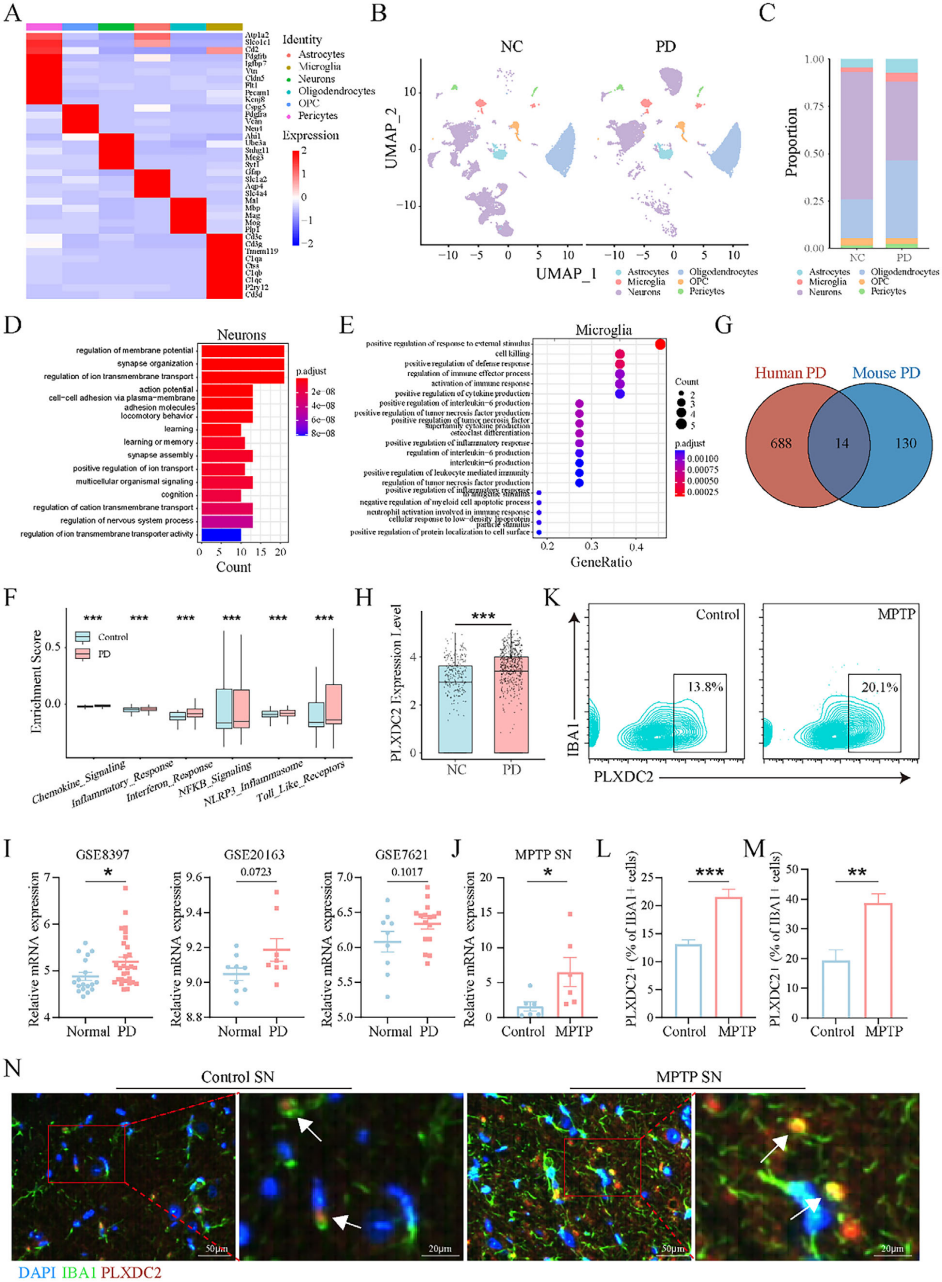
snRNA sequencing of the substantia nigra reveals high PLXDC2 expression in microglia. (A) Expression levels of canonical marker genes used to identify cell subtypes in the mouse substantia nigra; (B) UMAP plot showing clustering of substantia nigra cells from PD and control mice; (C) Distribution of cell proportions among different substantia nigra cell subpopulations in PD and NC mice; (D) GO enrichment analysis of highly expressed genes in neuronal subpopulations; (E) GO enrichment analysis of highly expressed genes in microglial subpopulations; (F) ssGSEA of inflammation‐related pathways in microglial subpopulations; (G) Venn diagram showing the overlap between highly expressed genes in microglial subpopulations from PD patients and those from PD mouse models; (H) PLXDC2 expression in microglial subpopulations of PD and control mice; (I) Differential expression analysis of PLXDC2 in the substantia nigra of PD patients and healthy controls based on three independent cohorts; GSE8397, *n* = 47; GSE20163, *n* = 17; GSE7621, *n* = 25. (J) qPCR analysis of PLXDC2 expression in the substantia nigra of PD and control mice (*n* = 6); (K) Representative flow cytometry plots showing PLXDC2 positive microglia in the mouse substantia nigra; (L) Flow cytometric quantification of PLXDC2 expression on IBA1 positive microglia isolated from the mouse substantia nigra (*n* = 6); (M) Immunofluorescence analysis of PLXDC2 expression in IBA1 positive microglia in the mouse substantia nigra (*n* = 6); (N) Representative immunofluorescence images showing the colocalization of PLXDC2 and IBA1 in the mouse substantia nigra. ^*^ *p* < 0.05, ^**^ *p* < 0.01, and ^***^ *p* < 0.001.

To explore how PD affects specific cellular subsets and their functional profiles within the substantia nigra, we utilized an MPTP induced mouse model. Following the confirmation of motor deficits through behavioral tests, substantia nigra tissues were harvested from both MPTP challenged and control groups for snRNA‐seq. After rigorous quality control, including the exclusion of low‐quality nuclei, potential doublets, and batch effects 27 685 high quality nuclei were preserved for subsequent investigation (Figure S8A,C). We employed UMAP for visualization and manually categorized the clusters using established mouse brain cell markers (Figure 2A) [23, 24]. Our analysis identified six primary cell populations: neurons, microglia, astrocytes, oligodendrocytes, oligodendrocyte precursor cells, and pericytes (Figure 2B and Figure S8D). In the MPTP group, neurons represented the predominant cell type, followed by oligodendrocytes, astrocytes, and microglia. Consistent with observations in the human substantia nigra, the proportion of neurons was significantly reduced in MPTP‐treated mice, whereas the proportion of microglia was markedly increased (Figure 2C). GO enrichment analysis of marker genes for neuronal subpopulations revealed significant associations with biological processes such as cognition, synapse assembly, and learning or memory, supporting the accuracy of our neuronal annotations (Figure 2D and Table S5). Considering the pivotal involvement of microglia in PD development, we next examined differentially upregulated genes in microglial subpopulations between MPTP‐treated and control mice and performed GO functional annotation. We observed significant activation of inflammation‐related pathways, including IL‐6 and TNF signaling, in microglia from MPTP‐treated mice (Figure 2E and Table S6). Consistently, single‐sample gene set enrichment analysis (ssGSEA) based on multiple inflammation related gene sets further confirmed the activation of inflammatory response associated signaling in microglia from MPTP‐treated mice (Figure 2F). To identify key genes commonly involved in PD onset and progression in both humans and mice, we intersected the differentially upregulated genes in microglia from PD patients and mouse. This integrative analysis yielded 14 genes that were consistently upregulated in the substantia nigra of both PD patients and PD mice, including PLXDC2, SLC38A2, and RAPGEF5 et al. (Figure 2G). Among these, we focused on PLXDC2, which has not been reported in PD. snRNA‐seq analysis demonstrated that PLXDC2 was significantly upregulated in microglia from the substantia nigra of MPTP‐treated mice (Figure 2H). We further analyzed three independent transcriptomic datasets of the human substantia nigra and consistently observed elevated PLXDC2 expression in PD patients compared with healthy controls (Figure 2I). qPCR analysis confirmed that MPTP treatment induced a significant upregulation of PLXDC2 expression in the mouse substantia nigra (Figure 2J). Subsequently, substantia nigra tissues from MPTP‐treated mice were dissociated into single‐cell suspensions, and flow cytometric analysis verified increased PLXDC2 expression in IBA1‐positive microglia from PD model mice (Figure 2K,L). Moreover, double immunofluorescence staining further confirmed the upregulation of PLXDC2 in microglia (Figure 2M,N). Notably, we also observed increased PLXDC2 expression in the substantia nigra of an AAV‐mediated human A53T α‐synuclein PD‐related rat model (Figure S9A). Collectively, analyses in both PD patients and PD mice demonstrate that PLXDC2 is overexpressed in microglia, suggesting a potential crucial role for PLXDC2 in PD onset and progression.

### 3.3 Construction and Characterization of HFn‐GM@siPLXDC2/DFO/CeO_2_‐NP

**FIGURE 3:**
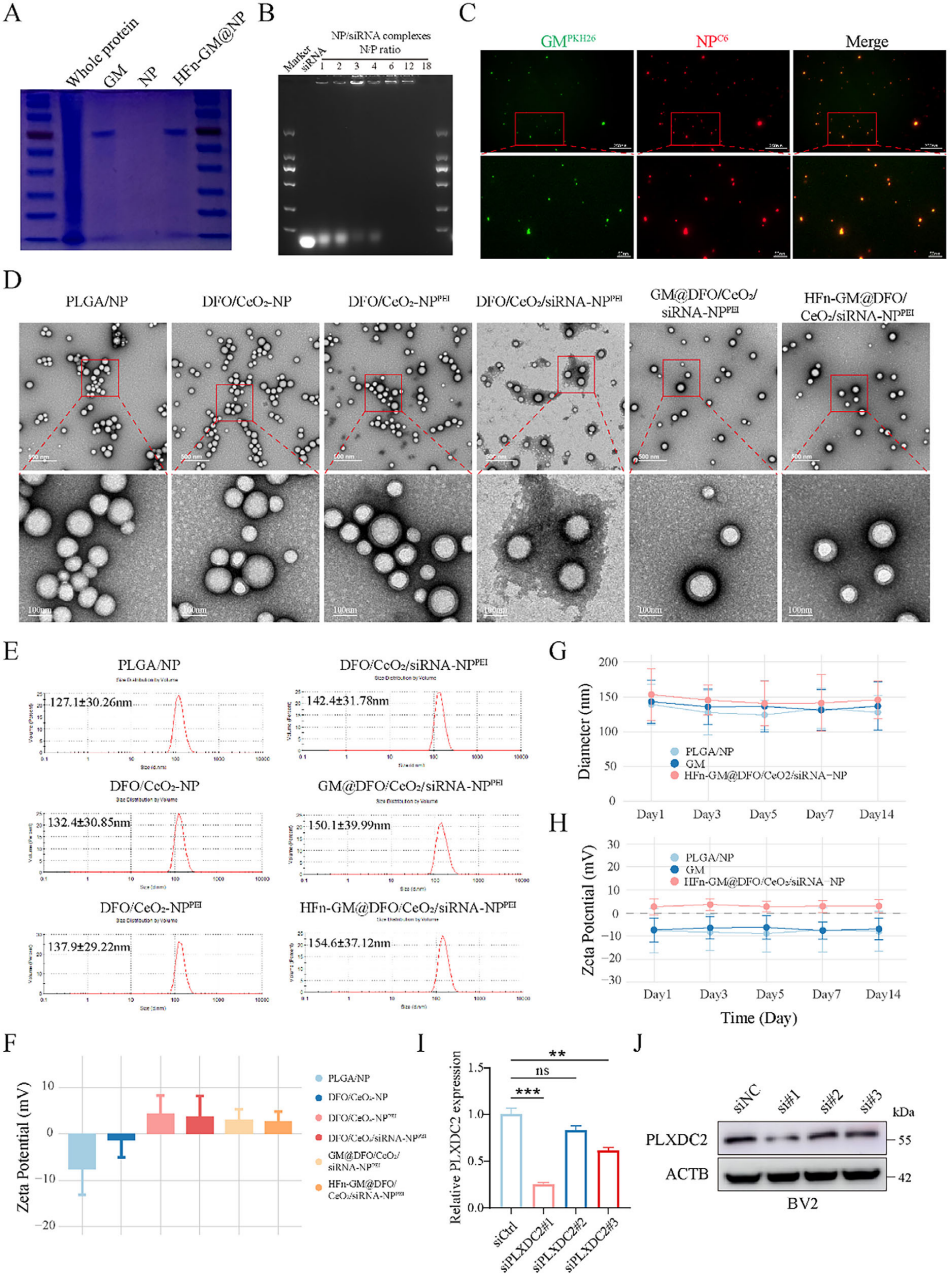
In vitro characterization of HFn‐GM@siPLXDC2/DFO/CeO_2_‐NP. (A) Coomassie Brilliant Blue staining of total proteins, GM, NP, and HFn‐GM@NP. (B) Formation of NP/siRNA complexes at different N/P ratios evaluated by agarose gel electrophoresis. (C) GM and NP were labeled with PKH26 and coumarin‐6, respectively, and analyzed by confocal fluorescence microscopy. (D) Representative transmission TEM images of different NP formulations. (E) Particle size distribution of different NP formulations. (F) Zeta potential of different NP formulations determined by dynamic light scattering. (G) Particle size stability of different NP formulations at the indicated time points. (H) Zeta potential stability of different NP formulations at the indicated time points. (I) Knockdown efficiency of different PLXDC2‐targeting siRNA sequences assessed by qPCR (*n* = 3). (J) Knockdown efficiency of different PLXDC2‐targeting siRNA sequences assessed by Western blot analysis. ^*^ *p* < 0.05, ^**^ *p* < 0.01, and ^***^ *p* < 0.001.

Given the role of PLXDC2 in the PD onset and progression, we developed a PLGA/NP siRNA delivery system to specifically target PLXDC2 in the substantia nigra. Accumulating evidence indicates that iron accumulation and excessive ROS generation in the substantia nigra are key pathological features of PD. Accordingly, the targeted delivery system was additionally loaded with the iron chelator DFO to alleviate iron deposition in the substantia nigra of PD mice [25], as well as CeO_2_ to suppress ROS generation [26, 27]. Although several chemically synthesized NP delivery platforms have shown promising therapeutic potential, their application in clinical settings is frequently constrained by intricate manufacturing processes and potential long‐term toxicity. In contrast, biomimetic vesicles exhibit superior biocompatibility and safety profiles. Our previous studies have demonstrated that microglial membrane coated NP display favorable BBB penetration and safety [13]. Moreover, recent reports indicate that HFn possesses substantial potential for targeted delivery in a variety of neurological disorders [28, 29, 30]. Briefly, DFO was first dissolved in deionized water to generate the inner aqueous phase, while PLGA/NP and CeO_2_ ultrafine NP were co‐dissolved in dichloromethane to form the oil phase. The aqueous phase was then emulsified into the oil phase, followed by a series of reactions to produce PLGA/NP loaded with DFO and CeO_2_ (DFO/CeO_2_‐NP). The NP were subsequently modified with PEI to confer a positive surface charge (DFO/CeO_2_‐NP^PEI^), enabling electrostatic adsorption of negatively charged siRNA (DFO/CeO_2_/siRNA‐NP^PEI^). GM were isolated from LPS‐stimulated microglia, mixed with DFO/CeO_2_/siRNA‐NP^PEI^, and repeatedly extruded to generate microglial membrane‐coated NP (GM@DFO/CeO_2_/siRNA‐NP^PEI^). To further obtain HFn‐modified biomimetic nanospheres (HFn‐GM@DFO/CeO_2_/siRNA‐NP^PEI^), the carboxyl groups on HFn were activated to form NHS esters and subsequently conjugated with DSPE‐PEG‐NH2 to generate DSPE‐PEG‐HFn. DSPE‐PEG‐HFn was then inserted into the lipid bilayer via hydrophobic interactions, yielding the final HFn‐GM@DFO/CeO_2_/siRNA‐NP^PEI^ formulation. Coomassie Brilliant Blue staining verified the successful isolation of microglial membranes (Figure 3A). Consistent with previous reports, gel retardation assays demonstrated a pronounced gel shift of siRNA encapsulated by PLGA/NP at an N/P ratio of 6:1 (Figure 3B). Microglial membranes and NP were labeled with PKH26 and C6, respectively, and fluorescence microscopy revealed substantial colocalization, indicating successful membrane coating of the NP (Figure 3C). TEM showed that both PLGA/NP and GM‐coated NP maintained intact vesicular structures with uniform spherical morphology (Figure 3D), with mean diameters of 127.1 and 147.2 nm, respectively (Figure 3E). Dynamic light scattering analysis revealed zeta potentials of −7.62 mV for PLGA/NP and −6.43 mV for GM (Figure 3F). Loading with DFO, CeO_2_, siRNA, and PEI modification did not markedly alter the morphology or size of PLGA/NP (Figure 3E,F). The particle sizes of DFO/CeO_2_‐NP, DFO/CeO_2_‐NP^PEI^, and DFO/CeO_2_/siRNA‐NP^PEI^ were 132.4, 137.9, and 142.4 nm, respectively (Figure 3E). PEI modification rendered the NP positively charged, resulting in increased surface potential: the zeta potentials of DFO/CeO_2_‐NP, DFO/CeO_2_‐NP^PEI^, and DFO/CeO_2_/siRNA‐NP^PEI^ were −1.38, 4.34, and 3.78 mV, respectively. The positively charged NP efficiently facilitated coating with negatively charged GM. TEM analysis further showed that both GM@DFO/CeO_2_/siRNA‐NP^PEI^ and HFn‐GM@DFO/CeO_2_/siRNA‐NP^PEI^ exhibited uniform morphology with clearly visible bilayer membrane structures, and their zeta potentials remained approximately 3 mV, indicating that HFn conjugation and GM coating did not substantially affect particle size or surface charge. Collectively, the TEM images and the modest changes in particle size and zeta potential across formulations confirmed the successful construction of DFO/CeO_2_/siRNA‐NP^PEI^ and the effective conjugation of HFn to the GM (Figure 3D–F). Furthermore, PLGA/NP, GM‐coated NP, and HFn‐GM@DFO/CeO_2_/siRNA‐NP^PEI^ maintained superior colloidal stability, as evidenced by the negligible fluctuations in hydrodynamic diameter and surface charge throughout a 14‐day incubation in PBS Figure 3G,H. Among the three candidate siRNA sequences designed to silence PLXDC2, in vitro screening identified siRNA #1 as the most potent candidate, yielding a markedly higher knockdown efficiency of PLXDC2 at both the transcript and protein levels in BV2 microglia (Figure 3I,J). Therefore, HFn‐GM@DFO/CeO_2_/siRNA‐NP^PEI^ carrying siRNA #1 was selected for subsequent therapeutic studies in PD.

### 3.4 Evaluation of BBB Permeability, Targeting Capability, and Biosafety of HFn‐GM@NP

**FIGURE 4:**
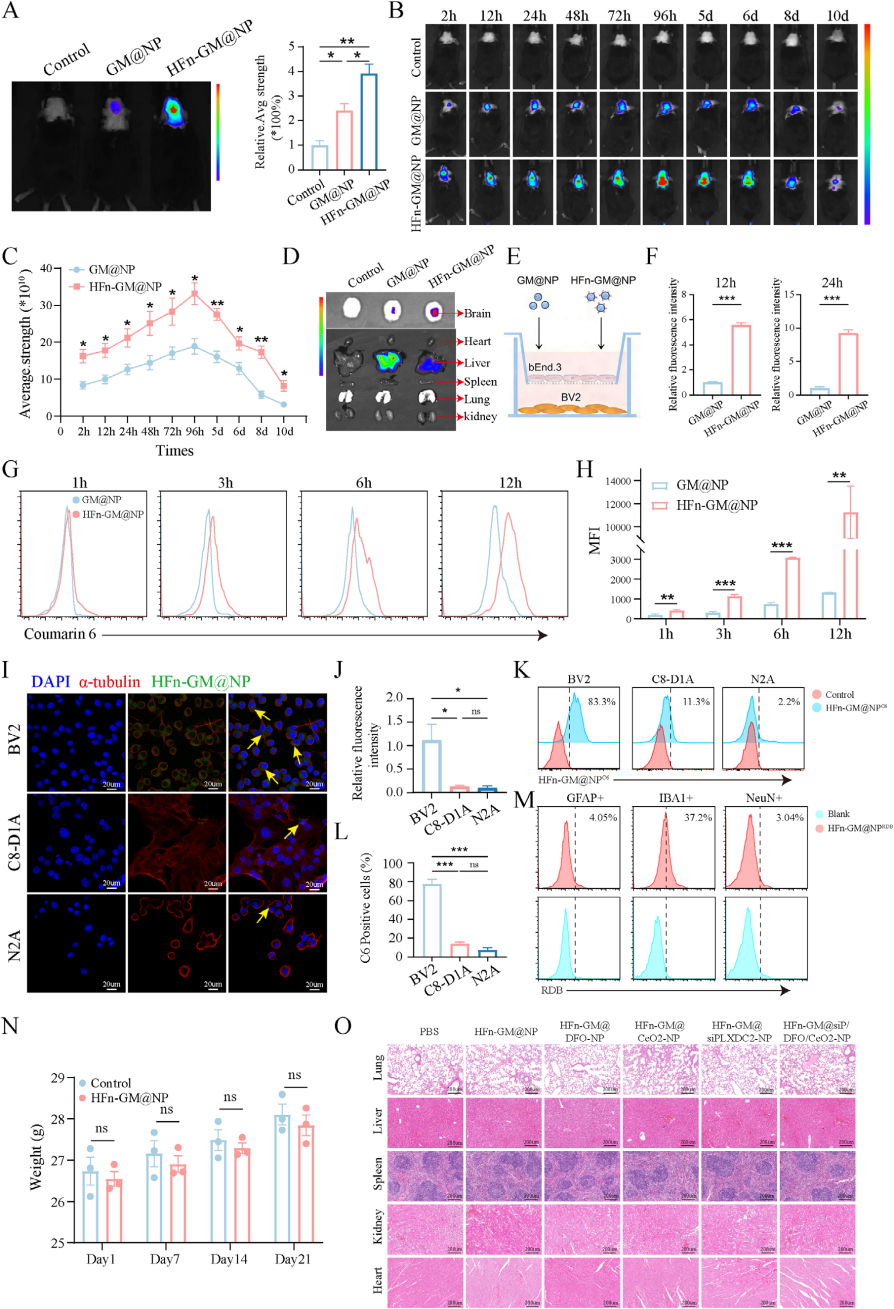
Evaluation of BBB penetration, lesion‐targeting capability, and biosafety of HFn‐GM@NP. (A) In vivo fluorescence imaging of substantia nigra accumulation following tail vein injection of GM@NP and HFn‐GM@NP (left), and corresponding quantitative analysis (right, *n* = 3). (B) Biodistribution of GM@NP and HFn‐GM@NP in the mouse brain at different time points. (C) Quantitative comparison of brain accumulation of GM@NP and HFn‐GM@NP at the indicated time points (*n* = 3). (D) Representative ex vivo fluorescence images of the mouse brain and major organs. (E) Schematic illustration of the in vitro BBB model. (F) Quantification of coumarin‐6 fluorescence intensity in BV2 cells after incubation for 12 and 24 h (*n* = 3). (G) Representative flow cytometry plots showing coumarin‐6 fluorescence intensity in BV2 cells incubated with GM@NP or HFn‐GM@NP for different durations. (H) Flow cytometric analysis of differences in coumarin‐6 fluorescence intensity in BV2 cells between the GM@NP and HFn‐GM@NP groups (*n* = 3). (I) Representative immunofluorescence images of BV2, C8‐D1A, and N2A cells internalizing HFn‐GM@NP. (J) Quantitative analysis of immunofluorescence intensity of HFn‐GM@NP^C6^ (*n* = 3). (K) Representative flow cytometry plots showing uptake of HFn‐GM@NP by BV2, C8‐D1A, and N2A cells. (L) Flow cytometric quantification of coumarin‐6 fluorescence intensity in BV2, C8‐D1A, and N2A cells (*n* = 3). (M) Representative flow cytometry plots showing uptake of HFn‐GM@NP_RDB by microglia, astrocytes, and neurons in vivo. (N) Body weight changes of mice treated with HFn‐GM@NP (*n* = 3). (O) Representative H&E‐stained images of major organs from mice receiving different treatments. ^*^ *p* < 0.05, ^**^ *p* < 0.01, and ^***^ *p* < 0.001.

The BBB is a critical physiological structure that separates the systemic circulation from the brain parenchyma and is essential for maintaining CNS homeostasis. Its highly selective permeability effectively restricts the entry of toxic substances into the brain but also substantially limits the central delivery efficiency and bioavailability of therapeutic agents for PD [31, 32, 33]. Considering that TFR1, the natural binding target of HFn, is upregulated in both BBB endothelial cells and disease‐associated cells within the substantia nigra of PD brains, we hypothesized that HFn modification of GM could enhance BBB penetration and targeting of pathological brain regions [14, 34, 35]. To test this hypothesis, DIR‐labeled GM@NP or HFn‐GM@NP was intravenously injected via the tail vein into MPTP‐induced PD model mice. 24 h after administration, in vivo small‐animal imaging was performed to monitor biodistribution. As depicted in Figure 4A, HFn‐GM@NP demonstrated superior accumulation in the lesioned brain regions compared to the GM@NP group, evidenced by a far more intense fluorescence signal. Furthermore, to elucidate the metabolic dynamics and intracerebral residence of the targeted NP, sequential imaging was executed at predefined time points after systemic injection (2, 12, 24, 48, 72, and 96 h, as well as days 5, 6, 8, and 10). Dynamic biodistribution analyses demonstrated that, at all‐time points examined, fluorescence in the HFn‐GM@NP group was markedly elevated relative to that in the GM@NP group, indicating that HFn modification markedly enhances BBB permeability and accumulation of GM@NP in brain lesion sites (Figure 4B,C). Ex vivo imaging of dissected major organs (including liver, spleen, lung, kidney and heart) and brain tissue further confirmed the enhanced brain‐targeted accumulation of HFn‐GM@NP compared with GM@NP (Figure 4D). Consistent with previous studies, the liver was identified as the primary metabolic organ for NP complexes (Figure 4D). Quantitative analysis of fluorescence intensity revealed that HFn modification significantly reduced hepatic accumulation of GM@NP. Although fluorescence signals in the kidney and spleen were higher in the HFn‐GM@NP group than in the GM@NP group, these differences were not statistically significant (Figure S9B,C)

To assess the BBB permeability of HFn‐GM@NP, we utilized an in vitro model based on the bEnd.3 cell line. These brain‐derived microvascular endothelial cells are characterized by their ability to establish a dense, confluent monolayer, making them a standard surrogate for the BBB [36, 37]. As shown in the Transwell co‐culture setup (Figure 4E), bEnd.3 cells occupied the apical inserts, whereas BV2 microglia were maintained in the basal compartments. After formation of a confluent bEnd.3 monolayer, C6‐labeled GM@NP or HFn‐GM@NP was added to the upper chamber. Uptake of NP by BV2 cells was assessed using fluorescence microscopy and flow cytometry. Fluorescence imaging showed that, after 12 and 24 h of co‐culture, BV2 cells in the lower chamber exhibited markedly stronger fluorescence signals following exposure to HFn‐GM@NP compared with GM@NP (Figure 4F and Figure S9D). Longitudinal flow cytometry data further confirmed that HFn‐GM@NP exhibited superior internalization by BV2 cells compared to the GM@NP group. Notably, this disparity in cellular accumulation became progressively more pronounced as the incubation period extended (Figure 4G,H). At 1 h, intracellular levels of HFn‐GM@NP in BV2 cells were 2.1‐fold higher than those of GM@NP, increasing to 8.4‐fold at 24 h (Figure 4H). We additionally compared the cellular uptake of GM@NP and HFn‐GM@NP in BV2 cells cultured alone. Flow cytometric analysis showed no significant difference in NP uptake between the two groups under the monoculture condition (Figure S9E). These findings indicate that HFn modification does not significantly enhance the direct uptake of NP by BV2 microglia. Together with the results from the BBB co‐culture model, these data suggest that the higher BV2‐associated uptake of HFn‐GM@NP following BBB translocation is more likely attributable to its enhanced BBB‐penetrating capacity rather than increased direct uptake by BV2 cells.

GM‐coated NP possess an inherent ability to target microglia, largely mediated by specific ligand‐receptor interactions on the microglial surface. Our previous work demonstrated that GM modified NP are preferentially taken up by microglial subpopulations [13]. To further assess the microglial targeting capability of HFn‐GM@NP, BV2 microglial cells, mouse neuroblastoma cells N2A, and mouse astrocyte cell line C8‐D1A were treated with C6‐labeled HFn‐GM@NP. Intracellular fluorescence was then evaluated by confocal laser scanning microscopy and flow cytometry. Fluorescence microscopy revealed a marked increase in fluorescent puncta distributed along the α‐tubulin cytoskeleton in BV2 cells treated with HFn‐GM@NP^C6^, whereas no significant differences were observed in N2A or C8‐D1A cells (Figure 4I,J). Flow cytometric analysis further confirmed a significantly higher C6 fluorescence intensity in BV2 cells following HFn‐GM@NP^C6 treatment (Figure 4K,L). Subsequently, in vivo cellular uptake analyses were performed using HFn‐GM@NP loaded with rhodamine B (RDB). Microglia, astrocytes, and neurons were identified by IBA1, GFAP, and NeuN staining, respectively, and RDB fluorescence intensity was quantified in single‐cell suspensions of substantia nigra tissue by flow cytometry. The results consistently demonstrated a higher level of HFn‐GM@NP accumulation in microglia compared with other cell types (Figure 4M and Figure S9E). Collectively, these in vitro and in vivo experiments further confirmed the microglia‐targeting capability of HFn‐GM@NP.

We next evaluated the biosafety of HFn‐GM@NP. One week after HFn‐GM@NP injection, peripheral blood samples were collected to assess potential effects on cardiopulmonary function, hepatic and renal function, and hematopoiesis. No significant alterations in liver and kidney function indices or hematological parameters were observed in HFn‐GM@NP treated mice compared with controls (Figure S10). In addition, HFn‐GM@NP administration did not induce abrupt changes in body weight (Figure 4N). Three weeks after treatment, major organs including the spleen, liver, heart, lung, and kidney were harvested for H&E staining. Histopathological analyses revealed no apparent abnormalities in tissues from mice treated with HFn‐GM@NP carrying CeO_2_, DFO, PLXDC2 siRNA, or the combined siPLXDC2/DFO/CeO_2_ formulation compared with controls, indicating that the HFn‐GM@NP delivery system exhibits favorable biocompatibility and safety profiles (Figure 4O).

### 3.5 HFn‐GM@siPLXDC2/DFO/CeO_2_‐NP Markedly Improved Cognitive and Motor Deficits in PD Mice

Motor and cognitive impairments are hallmark features of patients with PD as well as of PD model mice [2, 38]. As shown in Figure S11A–N, compared with non‐MPTP healthy control mice, MPTP/PBS mice exhibited impaired motor performance, reduced TH‐positive dopaminergic neurons, and decreased Nissl‐positive neurons at the therapeutic evaluation time point, confirming the presence of PD‐related behavioral and neuropathological alterations in the disease vehicle group. We next evaluated the therapeutic effects of HFn‐GM@siPLXDC2/DFO/CeO_2_‐NP on motor and cognitive behaviors in PD mice. In these therapeutic experiments, MPTP/PBS mice were used as the disease vehicle control for comparing the effects of different nano formulations. Non‐MPTP healthy control mice were analyzed separately as the normal baseline reference and compared with MPTP/PBS mice in Figure S11.

In addition to HFn‐GM@siPLXDC2/DFO/CeO_2_‐NP, several control formulations were prepared, including HFn‐GM@NP (lacking siPLXDC2, DFO, and CeO_2_), HFn‐GM@DFO‐NP (lacking siPLXDC2 and CeO_2_), HFn‐GM@CeO_2_‐NP (lacking siPLXDC2 and DFO), and HFn‐GM@siPLXDC2‐NP (lacking DFO and CeO_2_), which was used to compare the relative therapeutic contributions of DFO, CeO_2_, siPLXDC2, and the combined formulation.

**FIGURE 5:**
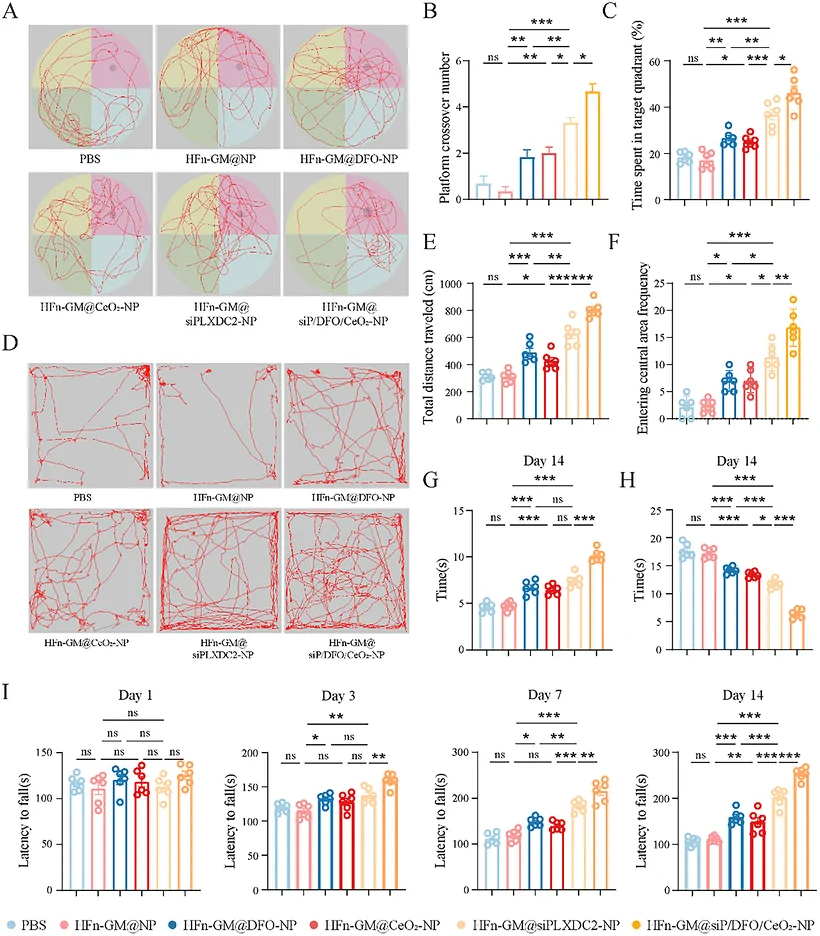
Assessment of cognitive and motor functions in PD mice. (A) Representative swimming trajectories of mice from different treatment groups in the Morris water maze test; (B) Statistical comparison of the number of platform crossings (*n* = 6); (C) Quantitative analysis of the relative percentage of time spent in the target quadrant (*n* = 6); (D) Representative locomotor trajectories of mice from different treatment groups in the open field test; (E) Quantification of the total distance traveled by mice (*n* = 6); (F) Statistical analysis of the number of entries into the central zone (*n* = 6); (G) Comparison of hanging time among mice from different treatment groups on day 14 of treatment (*n* = 6); (H) Statistical analysis of the time required for mice from different treatment groups to descend the pole on day 14 of treatment (*n* = 6); (I) Comparison of rotarod latency among mice from different treatment groups on days 1, 3, 7, and 14 of treatment (*n* = 6). ^*^ *p* < 0.05, ^**^ *p* < 0.01, and ^***^ *p* < 0.001.

Following intravenous administration of the different formulations, cognitive and motor functions of PD mice were evaluated at the indicated time points. Morris water maze test was used to assess spatial learning and memory. As shown in Figure 5A, MPTP/PBS mice and MPTP mice treated with empty HFn‐GM@NP exhibited random and unstable swimming trajectories and showed impaired performance in locating the target quadrant. Treatment with either DFO or CeO_2_ alone partially improved spatial memory performance. Notably, mice treated with HFn‐GM@siPLXDC2‐NP demonstrated superior spatial learning and memory compared with those receiving DFO or CeO_2_ alone. Importantly, mice treated with HFn‐GM@siPLXDC2/DFO/CeO_2_‐NP showed the most pronounced improvement in spatial memory. In terms of spatial memory enhancement, the group receiving combination therapy demonstrated a marked increase in both the frequency of platform crossings and the duration of stay within the target quadrant (averaging 42% of total time), significantly outperforming single‐agent cohorts (Figure 5B,C). To further evaluate spontaneous locomotion and anxiety‐related traits in PD mice under various conditions, an open field test was conducted. While MPTP‐induced mice tended to linger in the arena corners, those administered with HFn‐GM@siPLXDC2/DFO/CeO_2_‐NP displayed significantly reduced corner dwell time and more frequent transitions between central and peripheral zones, suggesting increased exploratory activity (Figure 5D). Further analysis of total distance and central area entries suggested that the nanoformulation effectively alleviated MPTP‐induced behavioral abnormalities (Figure 5E,F).

In addition, behavioral performance was further assessed using the traction test, vertical pole‐climbing test, and rotarod test. The traction test, which evaluates neuromuscular strength, showed that PD mice treated with HFn‐GM@siPLXDC2/DFO/CeO_2_‐NP exhibited a significantly prolonged hanging time compared with the empty HFn‐GM@NP control group, and this improvement increased with treatment duration (Figure 5G and Figure S12A). In the vertical pole‐climbing test, PD mice treated with HFn‐GM@siPLXDC2/DFO/CeO_2_‐NP treatment displayed a significantly reduced time to descend the pole, indicating improved motor coordination and balance (Figure 5H and Figure S12B). As shown in Figure 5I, MPTP‐induced neurotoxicity markedly shortened the latency to fall from the rotarod (<150 s), whereas HFn‐GM@siPLXDC2/DFO/CeO_2_‐NP treatment significantly prolonged rotarod performance time (>200 s). Together, these behavioral results indicate that DFO and CeO_2_ each conferred partial benefits in improving motor performance, whereas siPLXDC2 produced a more pronounced effect. Notably, the combined HFn‐GM@siPLXDC2/DFO/CeO_2_‐NP formulation achieved the greatest overall improvement, demonstrating its robust efficacy in restoring motor performance in PD mice.

### 3.6 HFn‐GM@siPLXDC2/DFO/CeO_2_‐NP Ameliorates Neuropathological Alterations in the Substantia Nigra of PD Mice

**FIGURE 6:**
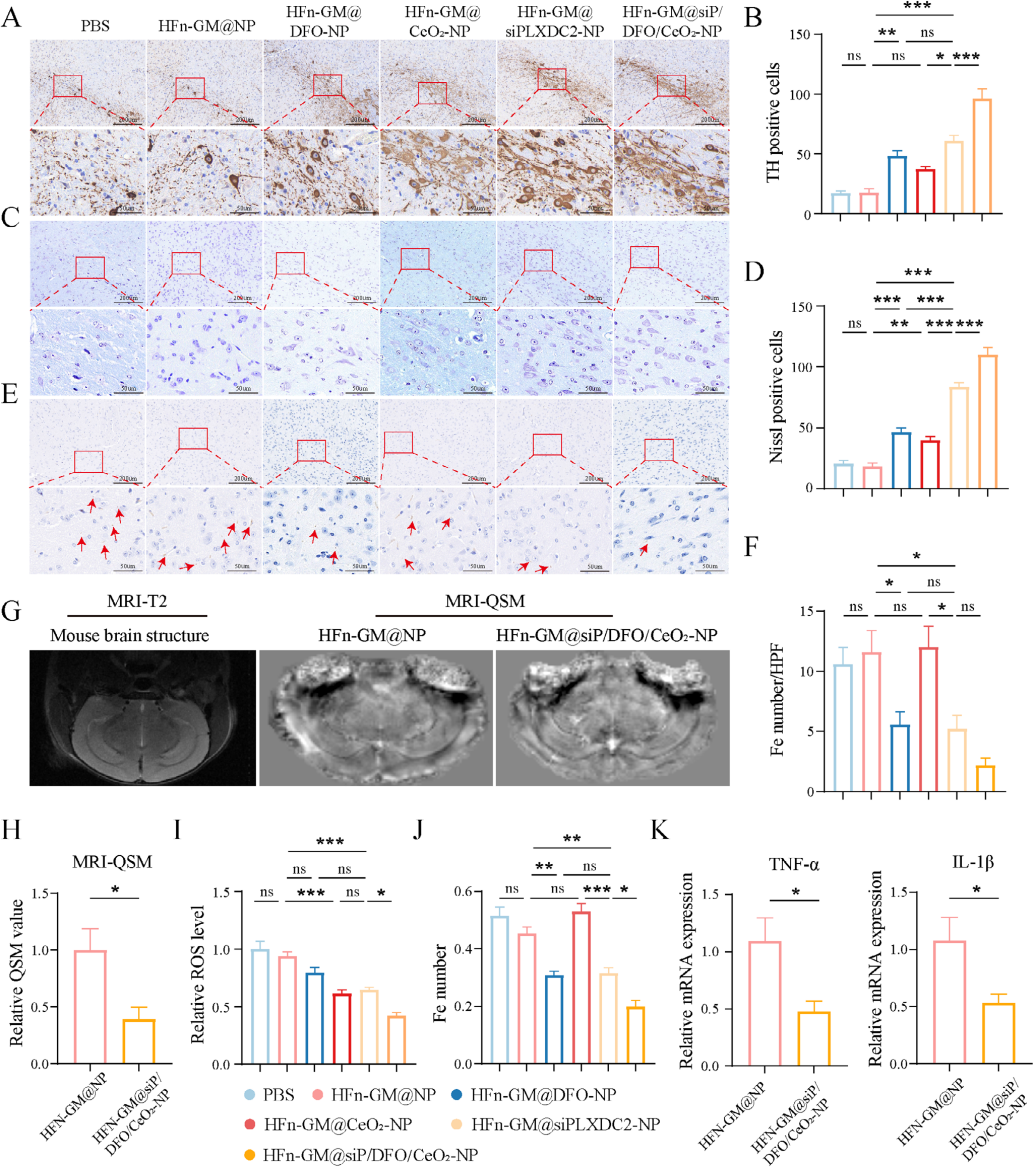
Pathological evaluation of therapeutic effects in PD mice. (A) Representative TH immunohistochemical staining images of the substantia nigra from mice in different treatment groups; (B) Quantitative analysis of TH‐positive cells (*n* = 6); (C) Representative Nissl staining images of the substantia nigra from mice in different treatment groups; (D) Quantification of Nissl body positive neurons (*n* = 6); (E) Representative Prussian blue staining images showing iron deposition in the substantia nigra from different treatment groups; (F) Quantitative analysis of iron ion content (*n* = 6); (G) Representative QSM images of the substantia nigra from mice treated with HFn‐GM@NP and HFn‐GM@siPLXDC2/DFO/CeO_2_‐NP; (H) Statistical analysis of QSM values in the substantia nigra of the HFn‐GM@NP and HFn‐GM@siPLXDC2/DFO/CeO_2_‐NP groups (*n* = 3); (I) ELISA‐based quantification of ROS levels in the mouse substantia nigra (*n* = 6); (J) ELISA‐based quantification of iron deposition levels in the mouse substantia nigra (*n* = 6); (K) qPCR analysis of neuroinflammatory cytokine expression in the mouse substantia nigra (*n* = 6). The same color coding was used consistently across all quantitative panels in Figure 6 to indicate the corresponding treatment groups. ^*^ *p* < 0.05, ^**^ *p* < 0.01, and ^***^ *p* < 0.001.

As the rate‐limiting catalyst for dopamine synthesis, tyrosine hydroxylase (TH) serves as a classic and definitive marker for identifying dopaminergic neurons. Diminished TH enzymatic activity and protein levels within the nigrostriatal pathway constitute core pathological features of PD [39, 40]. To quantify TH‐positive neurons and their expression profiles, we conducted immunohistochemical staining. Compared to control groups, mice receiving HFn‐GM@DFO‐NP, HFn‐GM@CeO_2_‐NP, and HFn‐GM@siPLXDC2‐NP showed elevated TH‐positive cell counts, with the siPLXDC2 cohort demonstrating the most striking improvement. Notably, the synergistic HFn‐GM@siPLXDC2/DFO/CeO_2_‐NP treatment led to a far more substantial recovery of TH‐positive neurons in the substantia nigra of PD models than any monotherapy (Figure 6A,B). Furthermore, Nissl staining indicated that while MPTP induction caused a severe loss of Nissl bodies in nigral neurons, HFn‐GM@siPLXDC2/DFO/CeO_2_‐NP administration effectively reversed this depletion (Figure 6C,D). Given that excessive iron accumulation in the substantia nigra is a well‐documented driver of PD progression [8, 41], we integrated the iron chelator DFO into our NP system to alleviate iron toxicity. Evaluation of iron deposition via Prussian blue staining confirmed that HFn‐GM@DFO‐NP treatment successfully lowered nigral iron concentrations in PD mice (Figure 6E,F). Intriguingly, mice treated with HFn‐GM@siPLXDC2‐NP also exhibited a significant reduction in nigral iron accumulation, and this effect was further enhanced in mice receiving HFn‐GM@siPLXDC2/DFO/CeO_2_‐NP treatment (Figure 6E,F). To further evaluate the impact of HFn‐GM@siPLXDC2/DFO/CeO_2_‐NP on iron deposition in vivo, QSM was performed using an 11.7 T small‐animal magnetic resonance imaging system. QSM analysis revealed that HFn‐GM@siPLXDC2/DFO/CeO_2_‐NP treatment significantly decreased QSM values in the substantia nigra of PD mice, indicating a substantial reduction in iron accumulation (Figure 6G,H). Recent studies have identified elevated levels of ROS and pro‐inflammatory mediators as critical pathological drivers and potential biomarkers of PD progression [42, 43]. Accordingly, substantia nigra tissues were collected to quantify ROS and iron levels. Our findings further demonstrated that HFn‐GM@CeO_2_‐NP reduced ROS levels and produced partial neuropathological improvement, supporting the antioxidant contribution of CeO_2_, while HFn‐GM@siPLXDC2/DFO/CeO_2_‐NP administration further lowered the ROS level, thereby alleviating the oxidative burden triggered by MPTP (Figure 6I). In parallel, this therapeutic approach successfully suppressed iron levels within the substantia nigra (Figure 6J). Given that pro‐inflammatory mediators such as IL‐1β, IL‐6, and TNF‐α are central to the pathogenesis and progression of PD, we quantified their expression levels. Results indicated that HFn‐GM@siPLXDC2/DFO/CeO_2_‐NP treatment significantly downregulated the expression of TNF‐α and IL‐1β in the nigral region, indicating a robust attenuation of neuroinflammatory responses (Figure 6K).

### 3.7 PLXDC2 Promotes Microglial Senescence and Facilitates the Pro‐Inflammatory Phenotype

**FIGURE 7:**
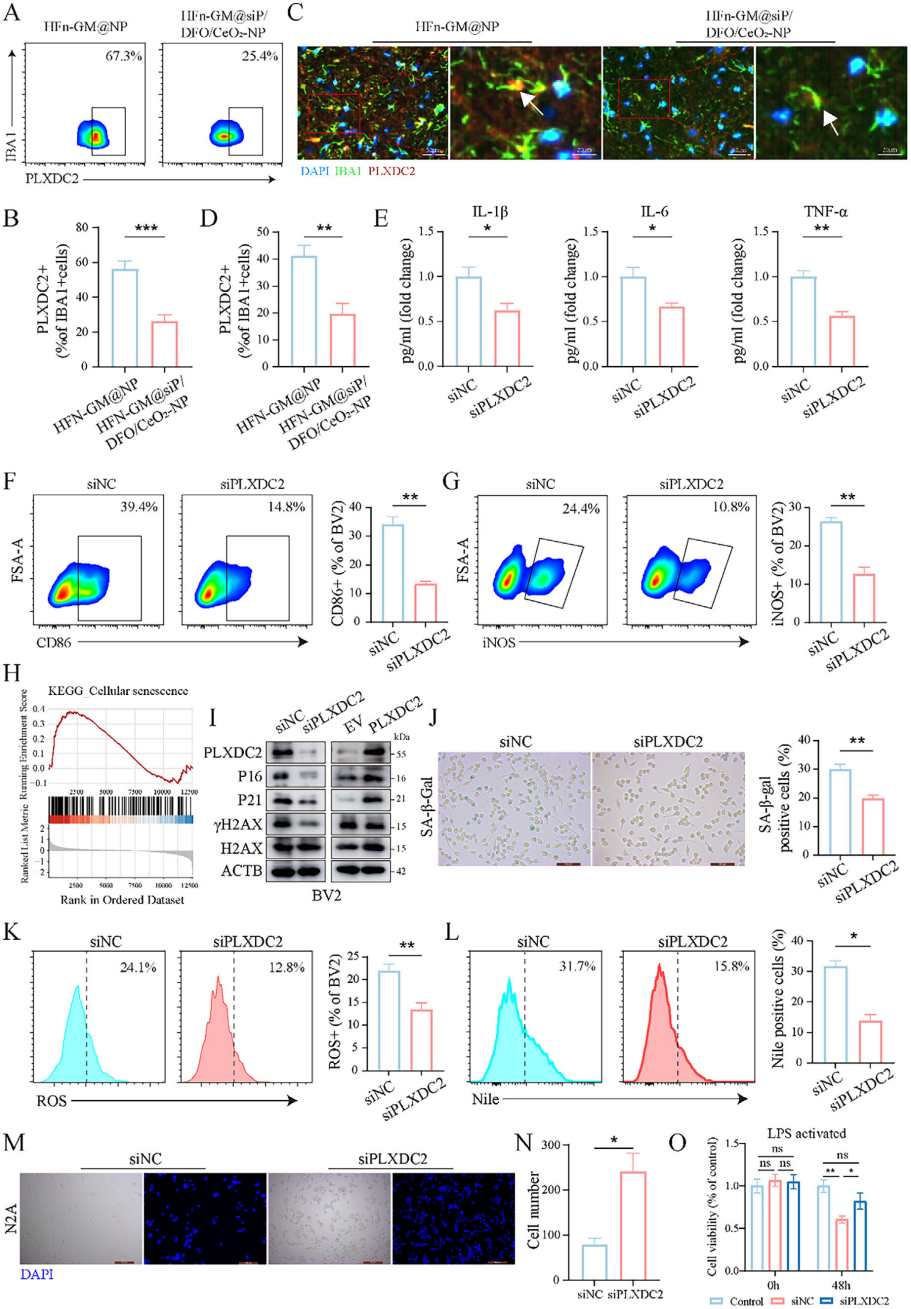
PLXDC2 induces microglial senescence and promotes the pro‐inflammatory phenotype. (A) Flow cytometric analysis of PLXDC2 expression in microglia isolated from the mouse substantia nigra; (B) Quantitative analysis of PLXDC2 expression levels in microglia from the mouse substantia nigra (*n* = 5); (C) Representative immunofluorescence images showing PLXDC2 and IBA1 expression in the mouse substantia nigra; (D) Quantitative comparison of the proportion of PLXDC2 positive microglia based on immunofluorescence analysis (*n* = 6); (E) ELISA quantification of neuroinflammatory cytokines in the supernatants of BV2 cells (*n* = 3); (F) Representative flow cytometric plots and quantitative analysis of the proportion of CD86 positive BV2 cells (*n* = 3); (G) Representative flow cytometric plots and quantitative analysis of the proportion of iNOS positive BV2 cells (*n* = 3); (H) GSEA demonstrating significant activation of cellular senescence related pathways in the PLXDC2 high expression group; (I) Western blot analysis of senescence‐associated protein expression in BV2 cells; (J) Representative images of SA‐β‐gal staining in BV2 cells and quantitative analysis of SA‐β‐gal positive cells (*n* = 3); (K) Representative flow cytometric plots and quantitative analysis of ROS‐positive BV2 cells (*n* = 3); (L) Representative flow cytometric plots and quantitative analysis of Nile Red positive BV2 cells (*n* = 3); (M) Representative fluorescence images of DAPI stained N2A cells in the BV2‐N2A co‐culture system; The BV2 cells were stimulated with LPS (1 µg/mL) for 24 h to induce an acute pro‐inflammatory state before co‐culture with N2A cells. (N) Quantitative comparison of DAPI‐positive cell numbers; (O) Assessment of N2A cell viability by CCK‐8 assay (*n* = 3). BV2 cells were acutely stimulated with LPS to induce a pro‐inflammatory state before co‐culture with N2A cells. Cell viability was normalized to the control group and expressed as a percentage. ^*^ *p* < 0.05, ^**^ *p* < 0.01, and ^***^ *p* < 0.001.

Based on behavioral assessments and neuropathological analyses, we observed that treatment with HFn‐GM@siPLXDC2‐NP yielded superior therapeutic efficacy compared with CeO_2_ or DFO monotherapy. Accordingly, the pronounced therapeutic benefit of the HFn‐GM@siPLXDC2/DFO/CeO_2_‐NP nanocomplex in PD mice is likely predominantly mediated by siPLXDC2, underscoring a critical role for microglial PLXDC2 in PD progression. To evaluate the regulatory role of PLXDC2 in PD progression, we first investigated whether HFn‐GM@siPLXDC2/DFO/CeO_2_‐NP could effectively modulate its expression within microglia. Microglial cells were isolated from the substantia nigra and analyzed via flow cytometry to determine the frequency of PLXDC2 positive cells. The results confirmed a substantial knockdown of PLXDC2 expression following treatment (Figure 7A,B). These findings were further supported by immunofluorescence co‐localization of IBA1 and PLXDC2, which demonstrated a marked reduction in PLXDC2 positive microglia within the substantia nigra of the nanomedicine‐treated group compared to HFn‐GM@NP controls (Figure 7C,D). To further elucidate the association between microglial PLXDC2 and PD pathogenesis, PLXDC2 was silenced in BV2 microglial cells. In line with the in vivo findings, PLXDC2 knockdown significantly reduced the mRNA levels of IL‐1β, IL‐6, and TNF‐α following LPS stimulation (Figure S13A). ELISA further confirmed a marked decrease in the secretion of IL‐1β, IL‐6, and TNF‐α in the culture supernatants (Figure 7E). Moreover, flow cytometric analysis demonstrated that PLXDC2 silencing effectively inhibited the polarization of BV2 cells toward a pro‐inflammatory phenotype, as evidenced by significantly reduced expression of the pro‐inflammatory markers CD86 and iNOS (Figure 7F,G). To further explore the biological functions regulated by PLXDC2, transcriptomic data from the substantia nigra of PD patients were stratified into low and high PLXDC2 expression groups, followed by GSEA to infer potential microglial functions associated with PLXDC2. Notably, KEGG based GSEA revealed that cellular senescence related signaling pathways were significantly enriched in samples with high PLXDC2 expression (Figure 7H).

Microglial function is critically dictated by both inflammatory states and cellular senescence. Under physiological conditions, microglia are essential for preserving central nervous system homeostasis, facilitating host defense, and supporting neuronal development [44, 45]. However, persistent and uncontrolled activation can trigger functional decline and the acquisition of a “senescent” phenotype [24]. Such senescent microglia typically display a senescence‐associated secretory phenotype (SASP), characterized by the release of diverse pro‐inflammatory cytokines, ROS, and other SASP factors. These components exert neurotoxic effects on the surrounding microenvironment, including neurons, thereby accelerating tissue dysfunction and disease progression [46, 47]. As established in previous literature, chronic stimulation of BV2 cells with LPS (500 ng/mL) for 10 days provides a reliable model for inducing cellular senescence [48, 49]. Prototypical molecular markers of this state include SA‐β‐gal activity and upregulated cyclin‐dependent kinase inhibitors such as p16 and p21. Additionally, elevated ROS levels and persistent DNA damage are recognized as key promoters of the senescent transition [50]. Notably, γ‐H2AX, a hallmark of DNA double‐strand breaks, plays a fundamental role in the recruitment of DNA repair machinery.

To further characterize the pro‐senescent role of PLXDC2, we examined its effects on this senescence associated markers. As shown in Figure 7I, PLXDC2 knockdown markedly suppressed p16 and p21 protein expression as well as H2AX phosphorylation, whereas PLXDC2 overexpression significantly enhanced p16/p21 expression and H2AX phosphorylation (Figure 7I and Figure S13B). SA‐β‐gal staining further demonstrated that PLXDC2 silencing substantially reduced SA‐β‐gal expression and enzymatic activity in BV2 cells (Figure 7J). Flow cytometric analysis revealed that PLXDC2 knockdown also significantly inhibited ROS generation in microglia (Figure 7K).

Previous studies have reported pronounced lipid droplet accumulation in microglia from aged mice and human brains, and such lipid droplet‐rich microglia exhibit characteristic pathological features, including elevated ROS production and increased secretion of pro‐inflammatory cytokines [51, 52]. Accordingly, Nile Red staining followed by flow cytometric analysis was performed to assess lipid droplet content in siNC and siPLXDC2 BV2 cells. Notably, the proportion of Nile Red positive cells was significantly lower in PLXDC2 silenced BV2 cells compared with controls (Figure 7L), indicating that reduced PLXDC2 expression suppresses ROS production and limits lipid droplet accumulation. Finally, a microglia‐neuron co‐culture system was established using N2A neuroblastoma cells to further investigate the impact of microglial PLXDC2 depletion on neuronal viability. The BV2 cells with different PLXDC2 expression were stimulated with LPS (1 µg/mL) for 24 h to induce an acute pro‐inflammatory state before co‐culture with N2A cells. DAPI staining and CCK‐8 assays revealed that conditioned medium derived from PLXDC2 silenced BV2 cells significantly promoted N2A cell viability compared with conditioned medium from PLXDC2 high BV2 cells (Figure 7M–O). These findings suggest that elevated PLXDC2 expression in microglia may exacerbate neuronal injury. Collectively, our results demonstrate that PLXDC2 promotes microglial senescence and contributes to the acquisition of a pro‐inflammatory phenotype, thereby contributing to PD pathogenesis.

### 3.8 PLXDC2 Promotes Microglial Senescence Through cGAS‐STING Signaling

**FIGURE 8:**
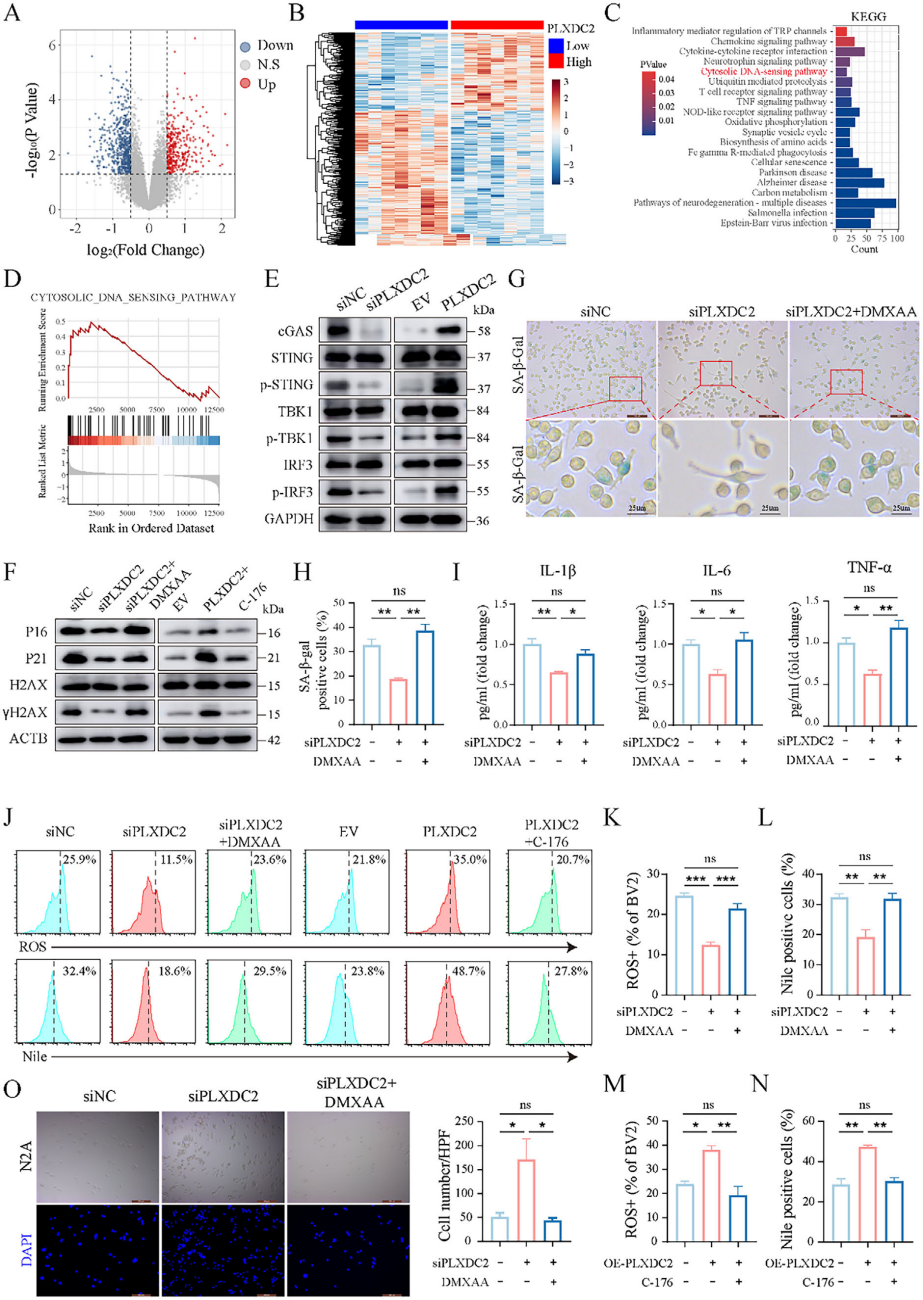
PLXDC2 promotes microglial senescence through cGAS‐STING signaling. (A) Volcano plot of differentially expressed genes between PLXDC2 high and low expression groups. (B) Heatmap showing differential gene expression between PLXDC2 high and low expression groups. (C) KEGG pathway enrichment analysis of differentially expressed genes. (D) GSEA enrichment analysis demonstrating significant activation of the CYTOSOLIC_DNA_SENSING_PATHWAY in the PLXDC2 high expression group. (E) Western blot analysis of proteins associated with the cGAS‐STING signaling pathway. (F) Western blot analysis of senescence‐related protein expression. (G) Representative images of SA‐β‐gal staining in BV2 cells. (H) Quantitative analysis of SA‐β‐gal positive cells (*n* = 3). (I) ELISA quantification of pro‐inflammatory cytokines in BV2 cell supernatants (*n* = 3). (J) Representative flow cytometry plots of ROS‐ and Nile Red positive BV2 cells. (K) Quantification of ROS‐positive microglia by flow cytometry following DMXAA treatment (*n* = 3). (L) Quantification of Nile Red positive microglia by flow cytometry following DMXAA treatment (*n* = 3). (M) Quantification of ROS positive microglia by flow cytometry following C‐176 treatment (*n* = 3). (N) Quantification of Nile Red positive microglia by flow cytometry following C‐176 treatment (*n* = 3). (O) Representative DAPI stained fluorescence images and quantification of N2A cells in the BV2‐N2A co‐culture system (*n* = 3). ^*^ *p* < 0.05, ^**^ *p* < 0.01, and ^***^ *p* < 0.001.

To elucidate the molecular pathways through which PLXDC2 modulates microglial senescence, we performed transcriptomic profiling on nigral samples from PD patients, stratified by PLXDC2 expression levels. Using the LIMMA package, we identified 325 significantly upregulated and 558 downregulated genes in the high PLXDC2 expression cohort (Figure 8A,B). Functional enrichment via GO and GSEA indicated that these DEGs were predominantly associated with inflammatory biological processes, including microglial activation and cytokine production (Figure S14A). KEGG pathway analysis further highlighted enrichment in pathways linked to PD, oxidative stress, and chemokine signaling, reinforcing the notion that PLXDC2 is a pivotal gene in PD progression. Interestingly, cellular senescence and the cytosolic DNA‐sensing pathway emerged as key signatures linked to PLXDC2 expression. GSEA confirmed significant activation of cytosolic DNA sensing in the PLXDC2‐high group, which was supported by GO analysis indicating a link between PLXDC2 and responses to double‐stranded DNA. Recent evidence has identified the cGAS‐STING axis as a promising therapeutic target in senescence‐driven neuroinflammation [53]. Wu et al. showed that the cGAS‐STING‐IRF3 signaling axis is a key pathway regulating hepatic stellate cell senescence [54]. Based on these observations, we hypothesized that PLXDC2 may promote the acquisition of a pro‐inflammatory senescent phenotype in microglia by modulating the cGAS‐STING signaling pathway. Western blot analyses showed that neither knockdown nor overexpression of PLXDC2 altered the total protein levels of STING, TBK1, or IRF3. However, PLXDC2 knockdown markedly reduced the phosphorylation levels of STING, TBK1, and IRF3, whereas PLXDC2 overexpression significantly enhanced the phosphorylation of these signaling proteins (Figure 8E and Figure S14B). In addition, a positive regulatory relationship between PLXDC2 and cGAS protein expression was observed (Figure 8E and Figure S14B).

To further verify whether PLXDC2 induces microglial senescence via the cGAS‐STING pathway, we examined senescence associated markers after pharmacological modulation of STING signaling. PLXDC2 knockdown significantly reduced p16 and p21 protein expression as well as H2AX phosphorylation in BV2 cells. Notably, treatment with the STING agonist DMXAA substantially reversed the reduction in p16/p21 expression and γH2AX levels induced by PLXDC2 silencing. Conversely, PLXDC2 overexpression markedly increased p16/p21 expression and γH2AX levels, whereas these effects were attenuated by the STING inhibitor C‐176 (Figure 8F and Figure S14C). Consistently, SA‐β‐gal staining showed that DMXAA restored the senescent phenotype suppressed by PLXDC2 knockdown, whereas C‐176 reduced the senescence‐associated changes induced by PLXDC2 overexpression (Figure 8G,H and Figure S14D). We next assessed the expression of neuroinflammatory cytokines in microglia using quantitative PCR and ELISA. DMXAA treatment increased the secretion of IL‐1β, IL‐6, and TNF‐α in siPLXDC2 BV2 cells, whereas C‐176 treatment reduced the secretion of these pro‐inflammatory cytokines in PLXDC2 overexpressing BV2 cells (Figure 8I and Figure S14E). Flow cytometric analyses further showed that DMXAA restored ROS production and lipid droplet accumulation after PLXDC2 knockdown, while C‐176 attenuated these phenotypes induced by PLXDC2 overexpression (Figure 8J–N). In addition, a BV2‐N2A transwell co‐culture system revealed that DMXAA abolished the proliferative advantage of N2A cells conferred by PLXDC2 knockdown in BV2 cells (Figure 8O). Collectively, these findings highlight the indispensable role of the cGAS‐STING signaling pathway in PLXDC2 induced microglial senescence and the consequent pro‐inflammatory phenotype.

Several limitations should be acknowledged in this study. First, although our transcriptomic analyses, in vivo siRNA mediated PLXDC2 suppression, and in vitro gain‐ and loss‐of‐function experiments collectively support a functional role of PLXDC2 in regulating microglial senescence, we have not yet established microglia‐specific PLXDC2 conditional knockout or overexpression mouse models. Therefore, the current data support PLXDC2 as a functional regulator and potential therapeutic target in PD associated microglial senescence, but do not fully establish its definitive causal role in vivo. In future studies, we will employ microglia‐specific genetic models, such as Cx3cr1‐CreER mediated PLXDC2 loss and gain‐of‐function systems, to further investigate the in vivo causal contribution of PLXDC2 to microglial senescence and PD progression. Second, the in vivo therapeutic experiments were primarily performed using the subacute MPTP‐induced PD mouse model. Although this model is widely used to evaluate dopaminergic neuronal injury, neuroinflammation, oxidative stress, iron deposition, and motor dysfunction, it does not fully recapitulate the long‐term disease progression and α‐synuclein aggregation observed in human PD. In addition, α‐synuclein pathology, including Lewy body‐like pathology, was not directly assessed in the present study. Therefore, the therapeutic effects observed should be interpreted in the context of MPTP‐induced dopaminergic neurotoxicity and inflammatory injury, rather than as direct evidence for modulation of α‐synuclein pathology. In future studies, we will further evaluate HFn‐GM@siPLXDC2/DFO/CeO_2_‐NP in AAV‐α‐synuclein, α‐synuclein PFF, genetically mediated, and other progressive α‐synuclein associated PD models.

## 4 Conclusions

In summary, we developed a multifunctional ferritin‐modified, microglial membrane‐coated nanoplatform, termed HFn‐GM@siPLXDC2/DFO/CeO_2_‐NP, for targeted and synergistic therapy of PD. We demonstrated that HFn modification markedly enhanced the ability of the biomimetic NP to traverse the BBB and selectively accumulate in diseased brain regions. By encapsulating the iron chelator deferoxamine and ultrasmall antioxidant CeO_2_ NP within the PLGA core, and electrostatically loading PLXDC2 targeting siRNA onto the NP surface, this nanoplatform simultaneously corrected iron dyshomeostasis, reduced reactive oxygen species generation, and suppressed the release of pro‐inflammatory cytokines. In vivo studies showed that this multi‐pronged intervention effectively protected dopaminergic neurons and significantly ameliorated motor dysfunction in PD mice. Importantly, we identified PLXDC2 as a critical regulator of microglial senescence in PD and demonstrated that its silencing attenuated neuroinflammation and oxidative stress via a cGAS‐STING dependent mechanism, thereby reducing neuronal injury. Collectively, our findings highlight a promising therapeutic strategy that integrates microenvironment‐responsive drug delivery with precise gene silencing to mitigate PD progression and restore motor function.

## Funding

This work was supported by the National Natural Science Foundation of China (Grant Nos. 82272571 and 82372418); the Peking University Clinical Scientist Training Program (BMU2025PYJH04); the Jiangsu Provincial Health Commission (Grant No. BJ23030); and the Jiangsu Provincial Research Hospital (Grant No. YJXYY202204‐YSB73).

## Ethics Statement

All animal procedures were conducted in strict accordance with the experimental protocols approved by the Animal Ethics Committee of Nantong University (Approval No. S20230228‐201).

## Conflicts of Interest

The authors declare no conflicts of interest.

## Supporting information




**Supporting File 1**: advs77793‐sup‐0001‐SuppMat.pdf.

**Supporting File 2**: advs77793‐sup‐0002‐SuppMat.xlsx.

## Data Availability

The data that support the findings of this study are available from the corresponding author upon reasonable request.
