# Supplementary material for: PLXDC2 siRNA‐Mediated Intervention Attenuates Microglial Senescence Through cGAS‐STING Signaling
Source: Adv Sci (Weinh). 2026 Sep 27:e77793. Online ahead of print. doi: 10.1002/advs.77793 (PMC13616238; doi:10.1002/advs.77793)
Supplement: Supplementary file 1 [file ADVS-9999-e77793-s002.pdf]

Supporting Information

PLXDC2 siRNA-Mediated Intervention Attenuates Microglial Senescence Through cGAS-STING Signaling

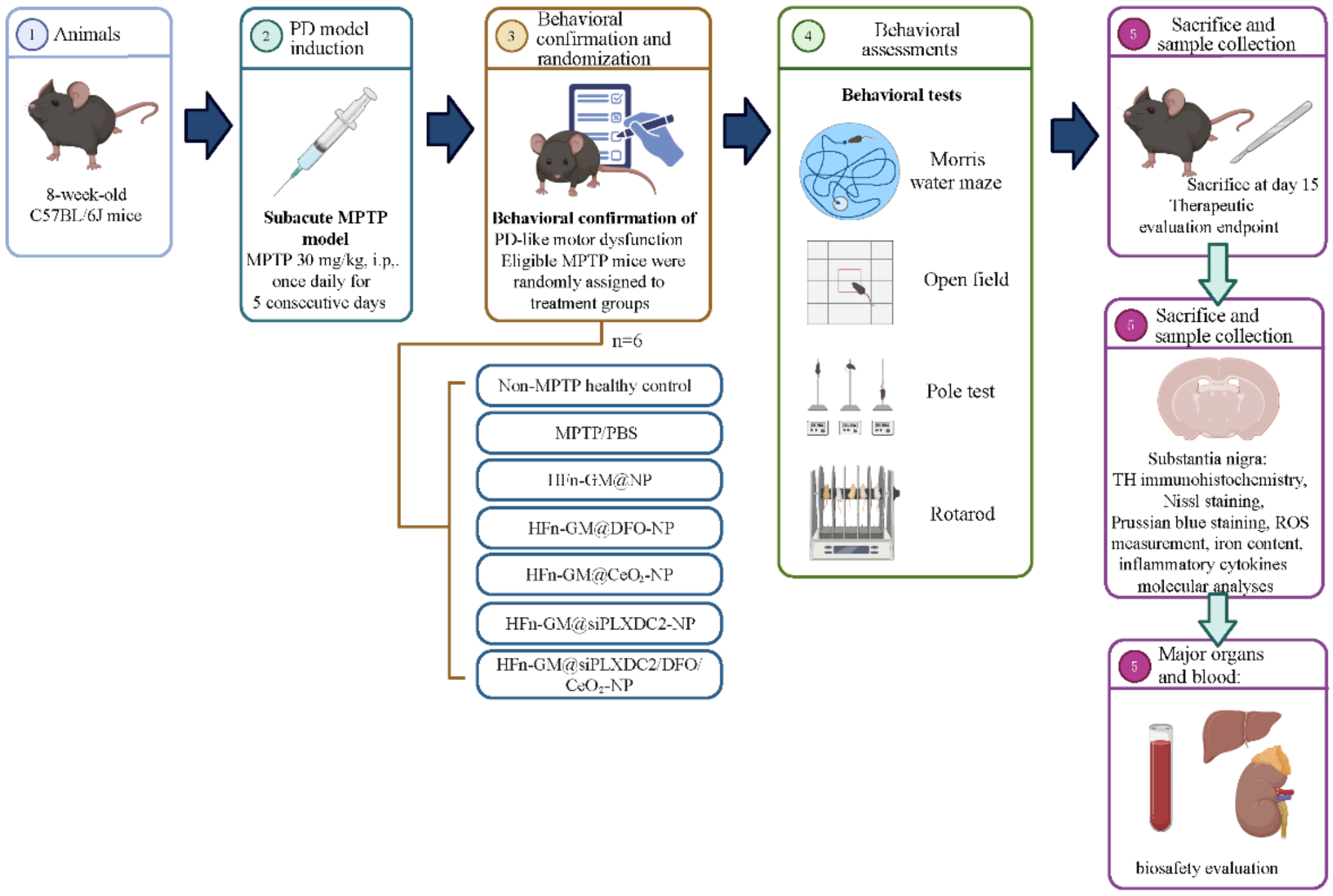

Figure S1. The overview diagram of the animal study design in this study.

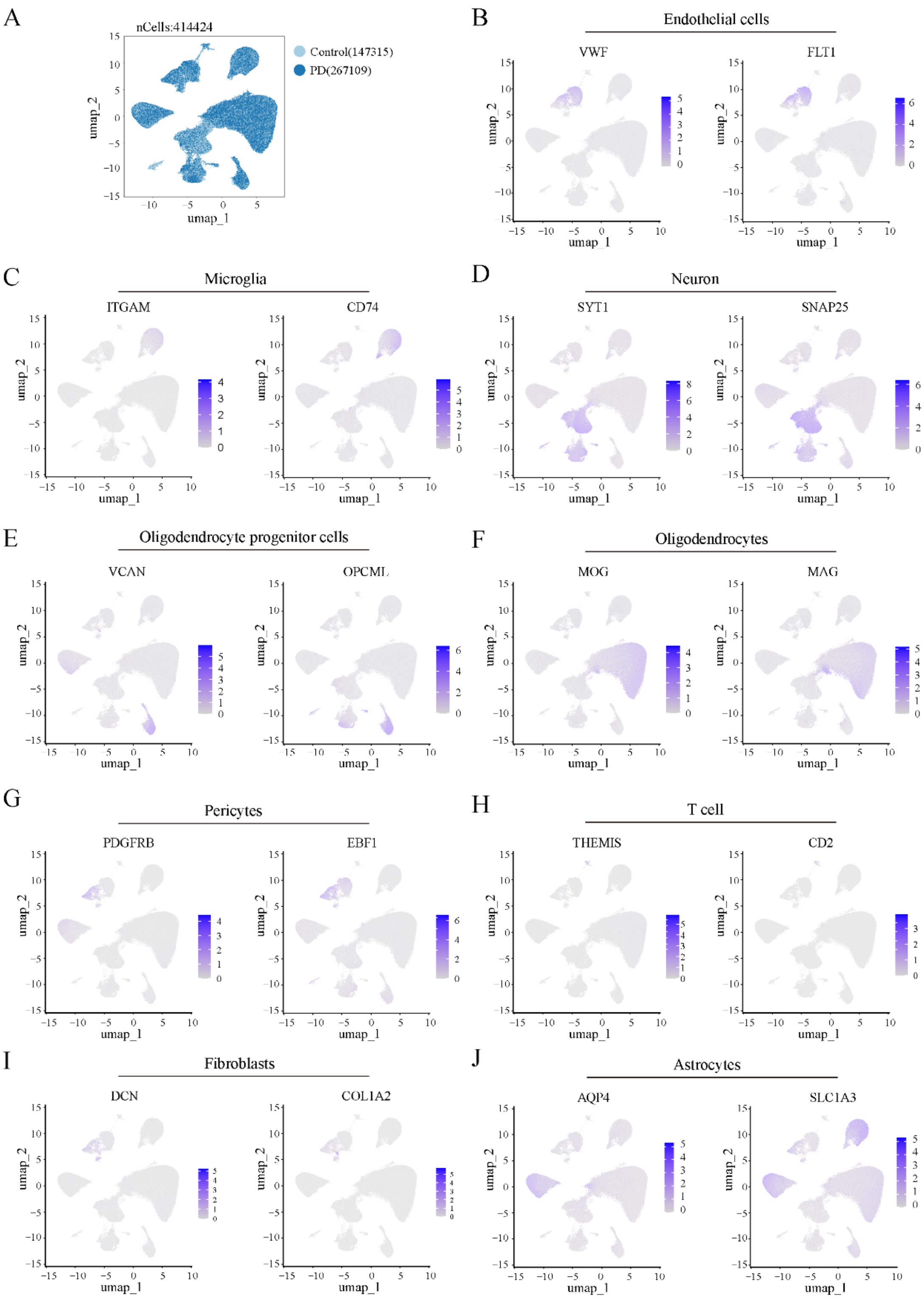

Figure S2. Expression profiles of cell type specific markers used to identify cellular subtypes in the human substantia nigra. (A) UMAP visualization of cell clusters after Harmony based batch correction; **(B-J)** UMAP plots showing the expression levels of canonical marker genes defining distinct cell types in the human substantia nigra.

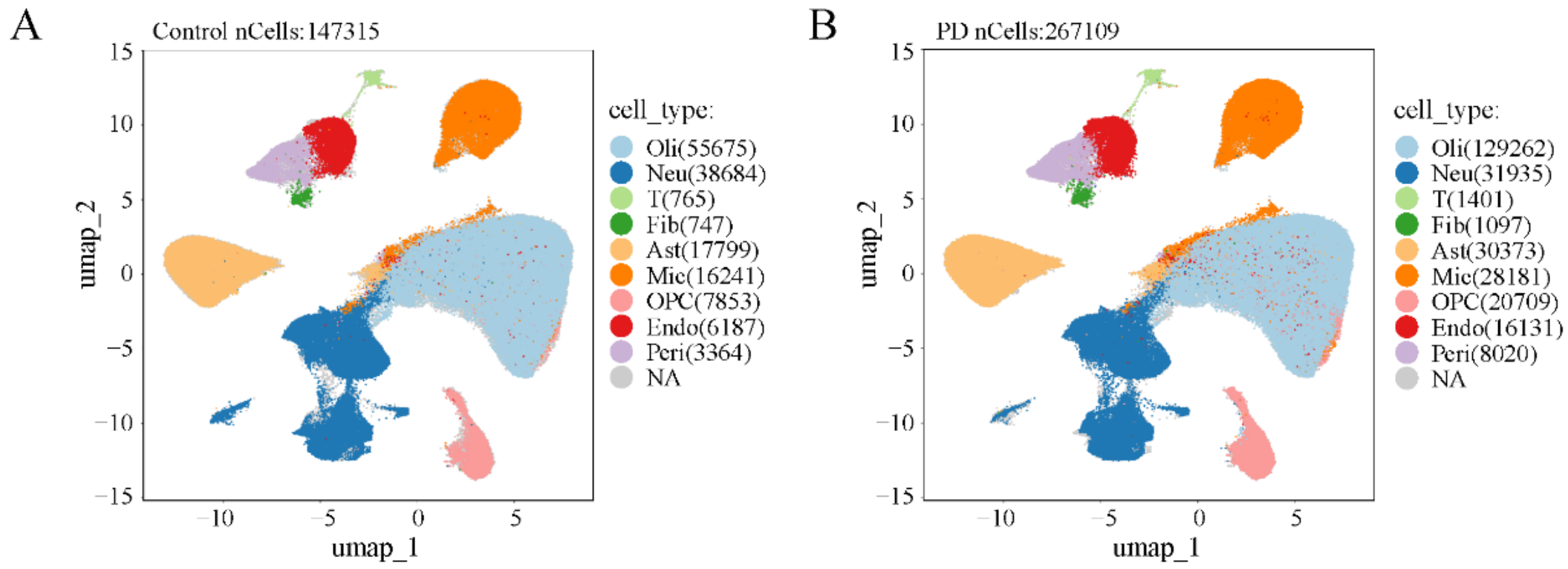

Figure S3. UMAP clustering analysis of snRNA-seq data from the substantia nigra of PD patients. (A) UMAP clustering of substantia nigra cells from healthy controls; **(B)** UMAP clustering of substantia nigra cells from patients with PD.

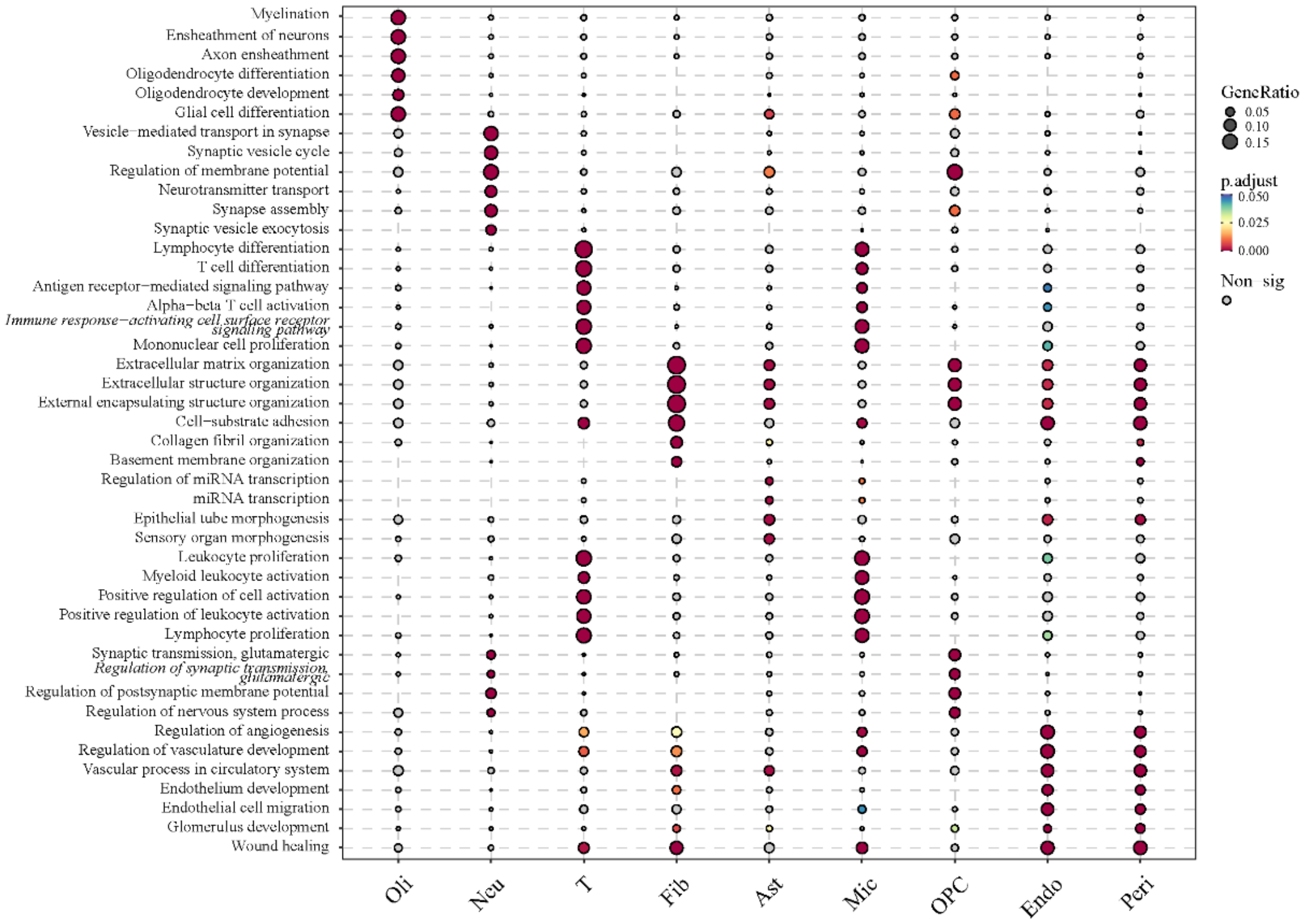

Figure S4. GO functional enrichment analysis of marker genes identified for each substantia nigra cell subpopulation.

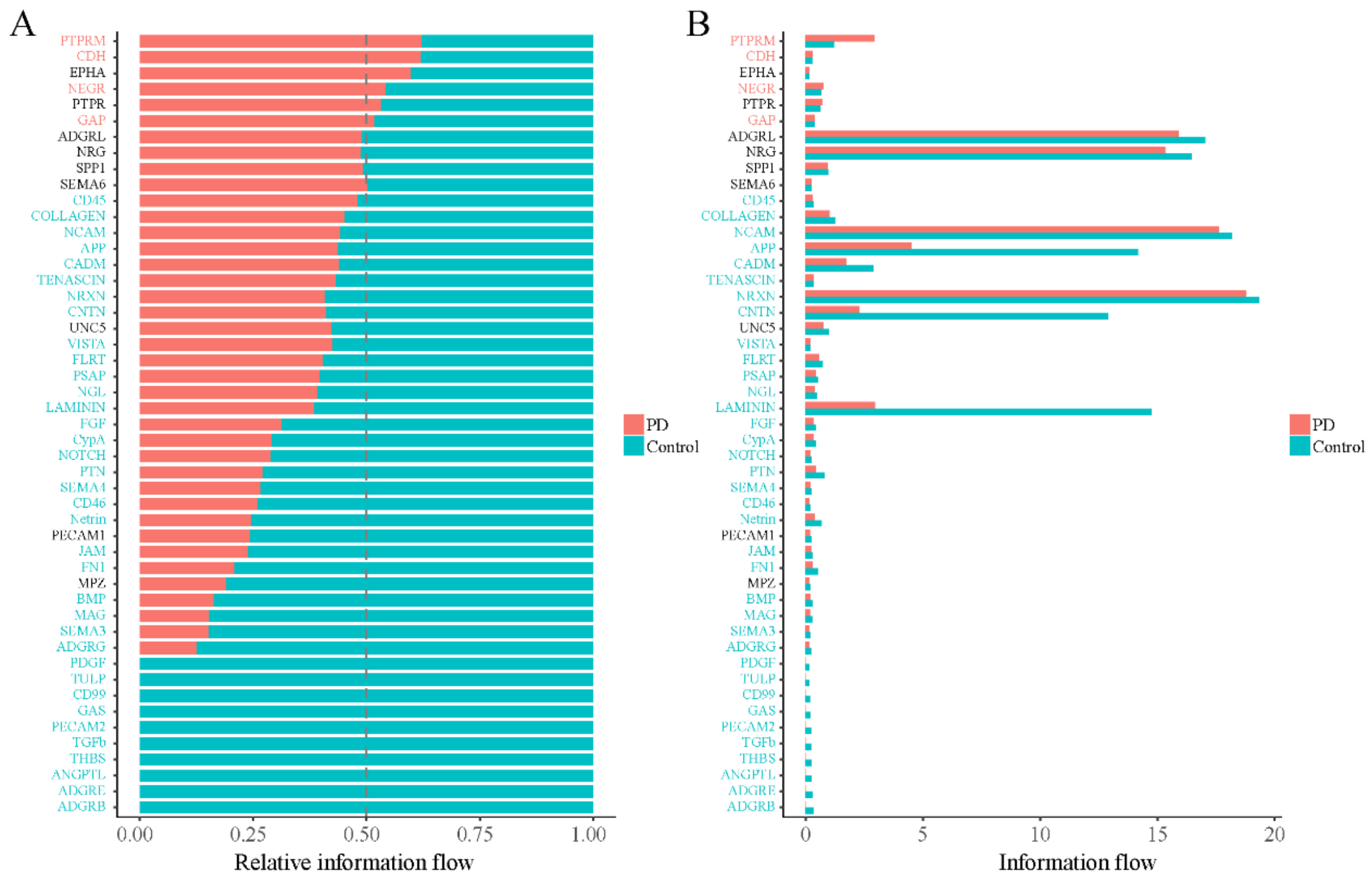

Figure S5. Alterations in information flow of major signaling pathways between PD and control groups. (A) Differential proportion plot illustrating changes in major signaling pathways; **(B)** Bar plot depicting differences in information flow across major signaling pathways.

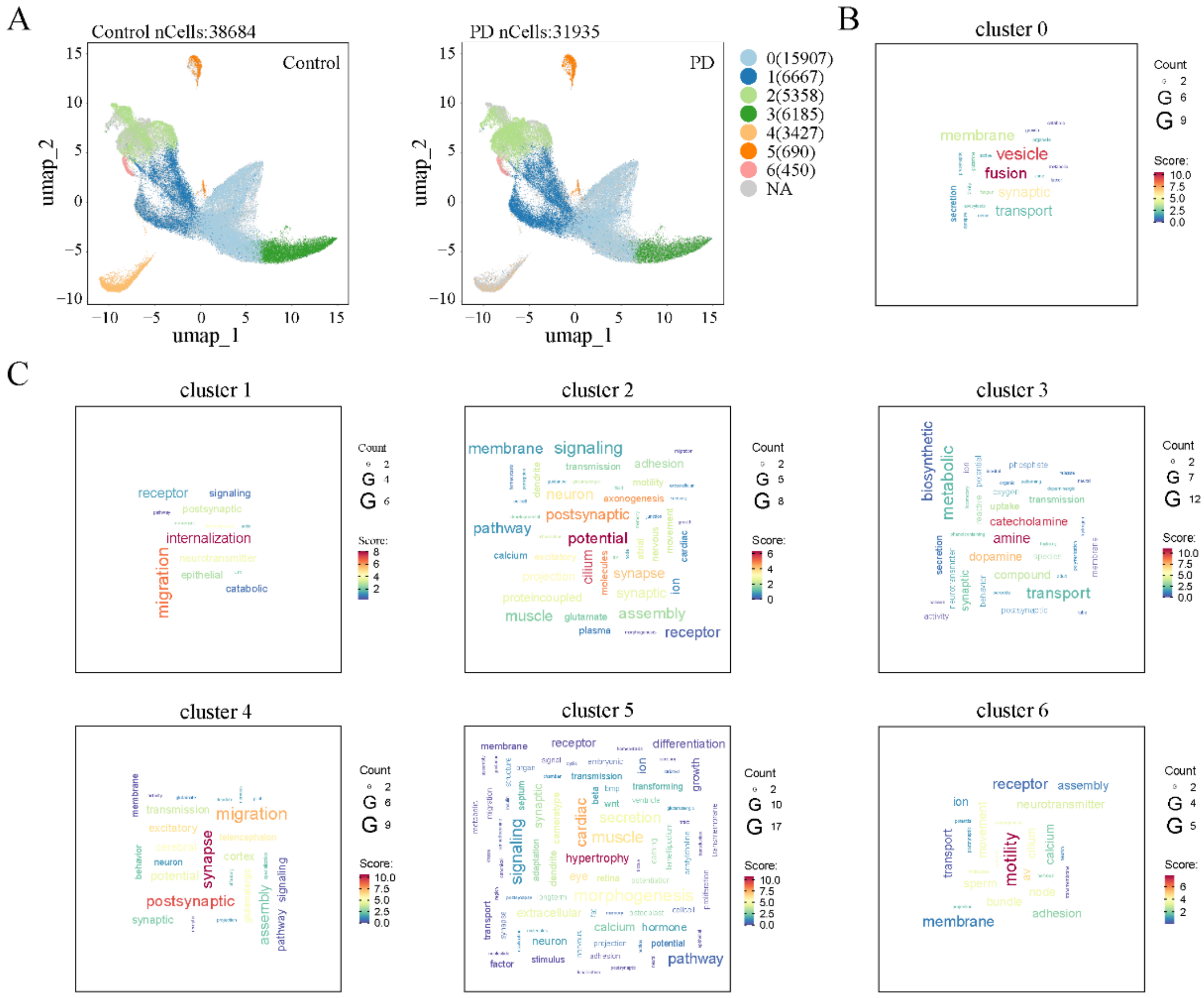

Figure S6. Functional enrichment analysis of identified neuronal cell clusters. (A) UMAP clustering of neuronal subpopulations in the substantia nigra from PD patients and controls; (B-C) Visualization of GO enrichment analyses for marker genes of each neuronal cell cluster.

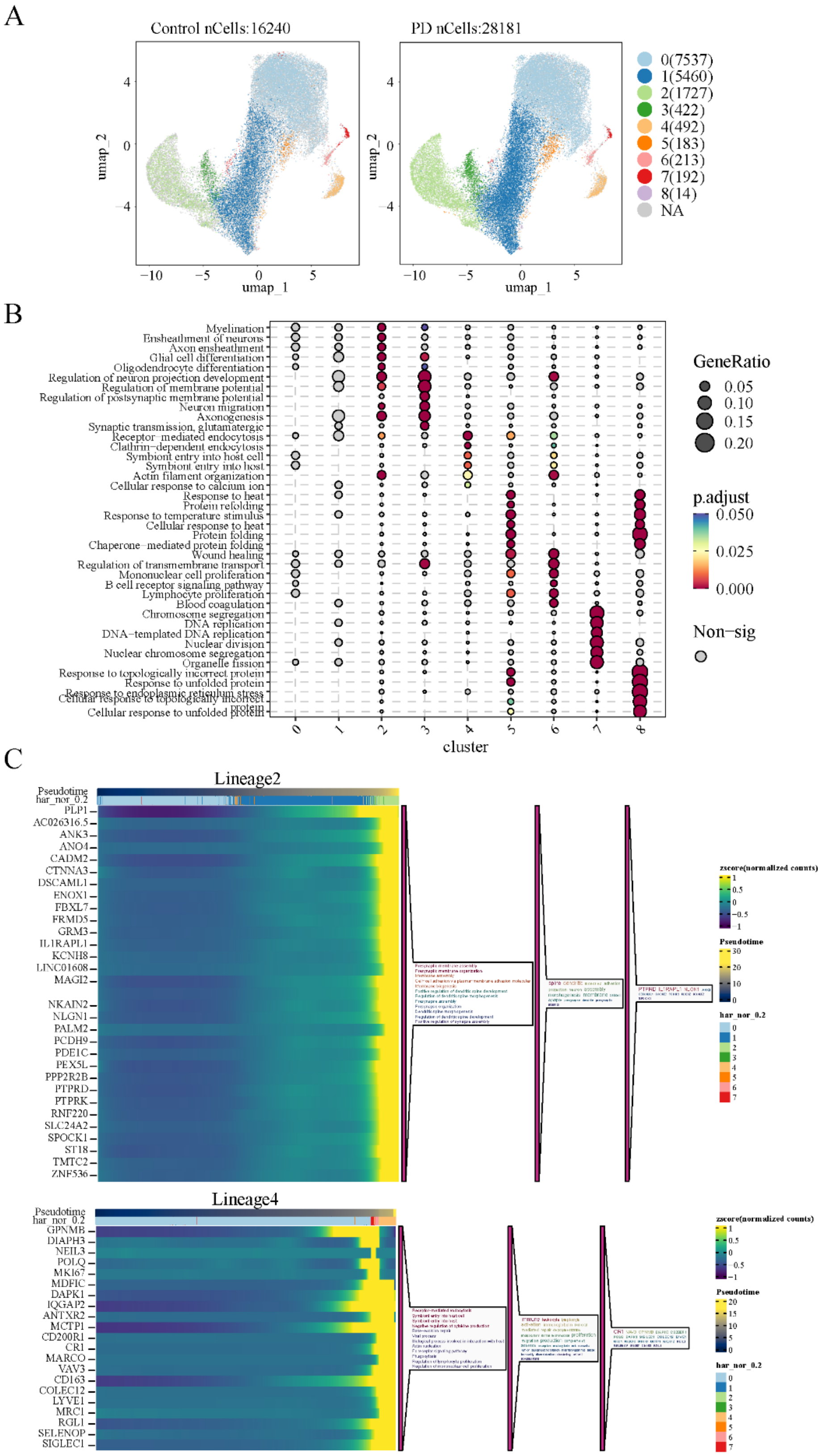

Figure S7. Pseudotime trajectory analysis of microglial cells. (A) UMAP clustering of microglial subpopulations in the substantia nigra from PD patients and healthy

controls. **(B)** Visualization of GO enrichment analysis of marker genes across distinct microglial clusters. **(C)** Heatmaps depict dynamic gene expression changes along pseudotime in Lineage 2 (upper panel) and Lineage 4 (lower panel), as inferred by pseudotime trajectory analysis, and GO enrichment analysis for these genes.

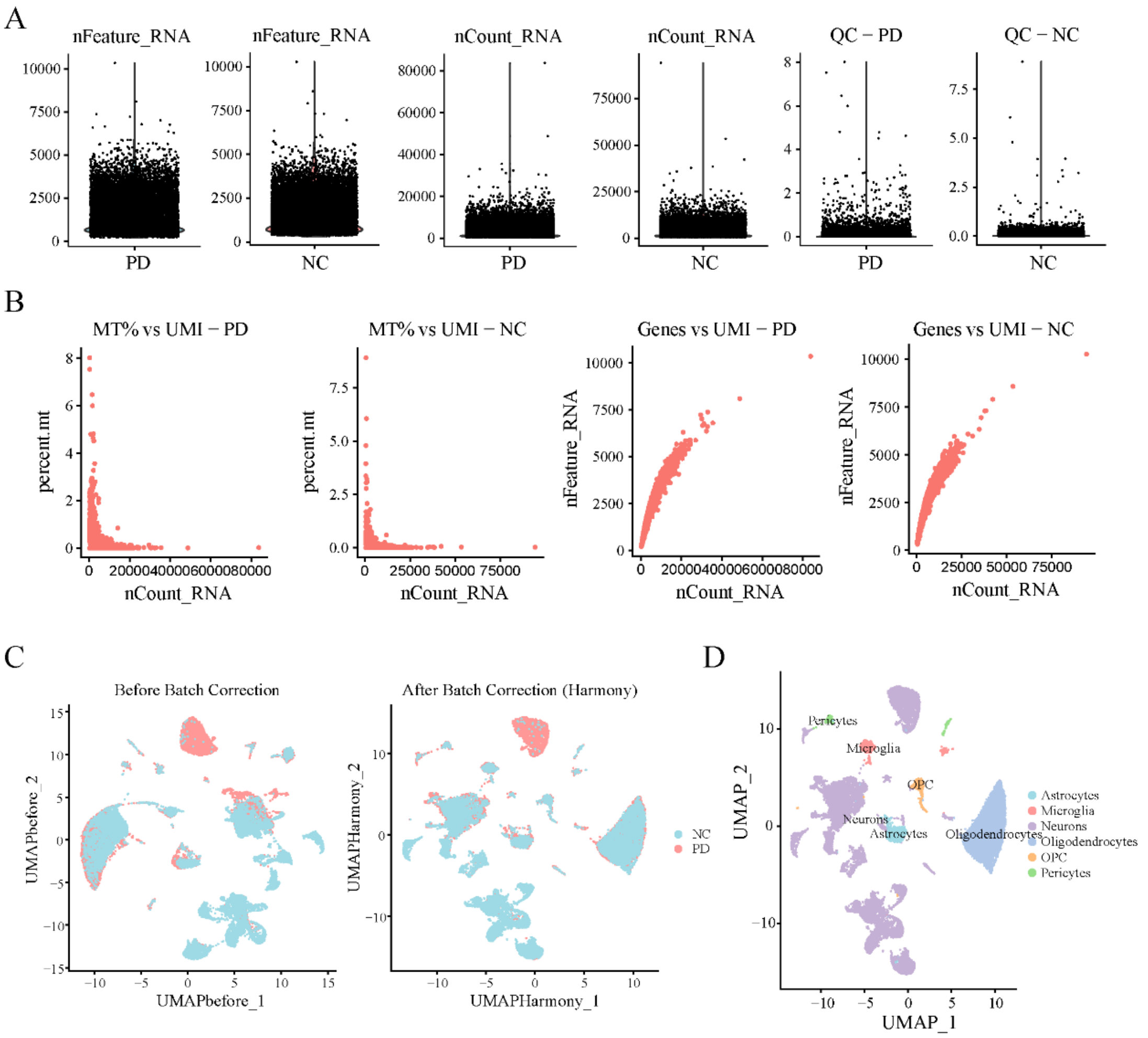

Figure S8. Quality control of snRNA sequencing data from the substantia nigra of PD mouse. (A) Violin plots showing the distribution of the number of detected genes per nucleus (nFeature_RNA), total UMI counts (nCount_RNA), and mitochondrial gene percentage (percent.mt) in PD and NC samples. (B) Scatter plots illustrating the relationships between mitochondrial gene percentage and UMI counts (percent.mt vs. nCount_RNA), as well as between the number of detected genes and UMI counts (nFeature_RNA vs. nCount_RNA) in PD and NC samples. (C) UMAP plots before and after batch correction based on sample classification. (D) UMAP clustering analysis of substantia nigra cells from PD mice, in which six distinct cell subpopulations were identified.

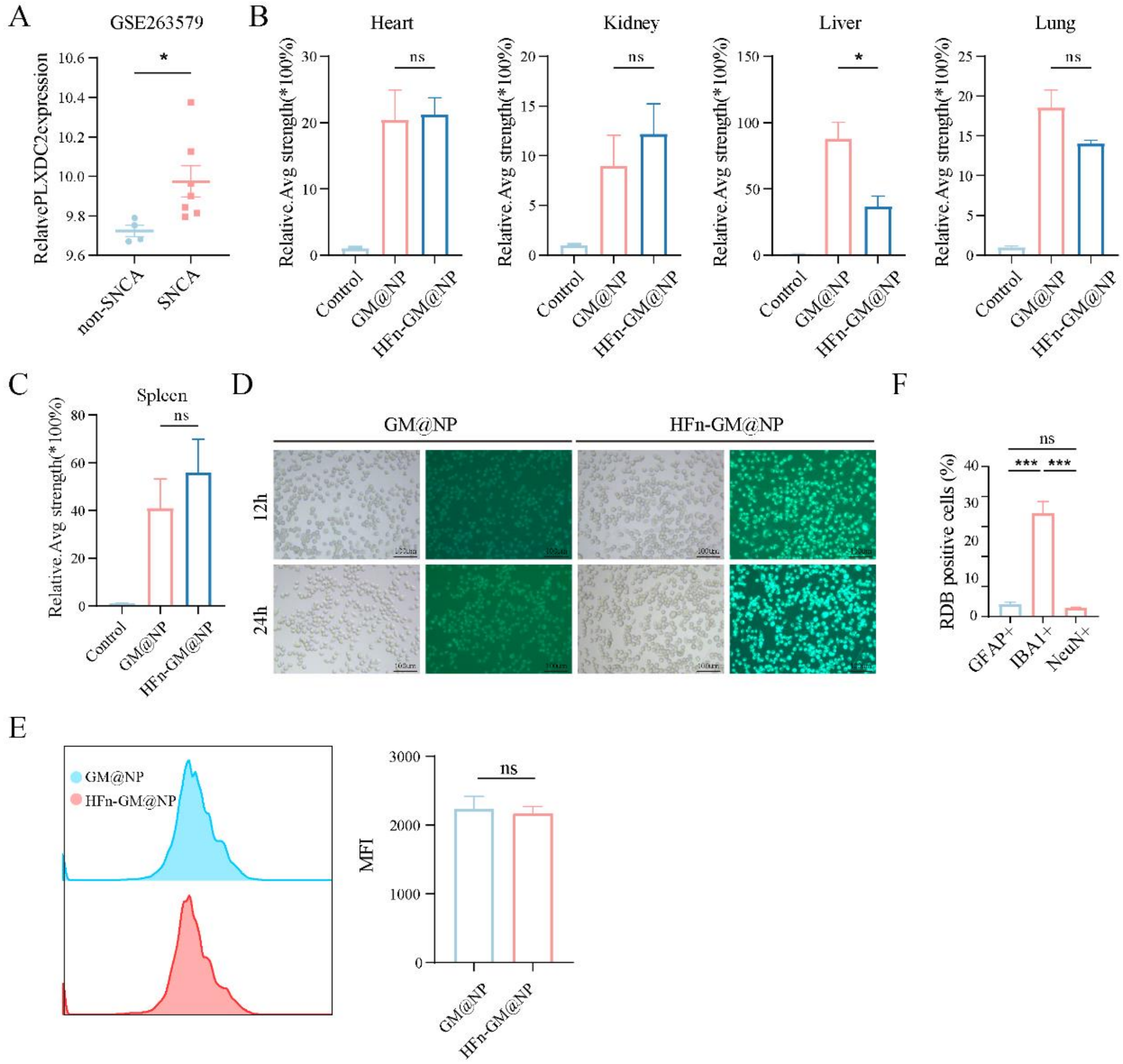

Figure S9. Targeting capability and biosafety evaluation of HFn-GM@NP. (A) PLXDC2 expression in the SN of an AAV-mediated human A53T α-synuclein PD-related rat model from GSE263579 (n=11). **(B-C)** Biodistribution of GM@NP and HFn-GM@NP in major organs of mice (n=3). **(D)** Representative fluorescence images of BV2 cells treated with GM@NP^{C6}, and HFn-GM@NP^{C6}. **(E)** Cellular uptake of GM@NP and HFn-GM@NP by BV2 microglia cultured alone was analyzed by flow cytometry (n=3). **(F)** Flow cytometric quantification of fluorescence intensity of HFn-GM@NP^{RDB} in microglia, astrocytes, and neurons in vivo (n=3). *P < 0.05, **P < 0.01, and ***P < 0.001

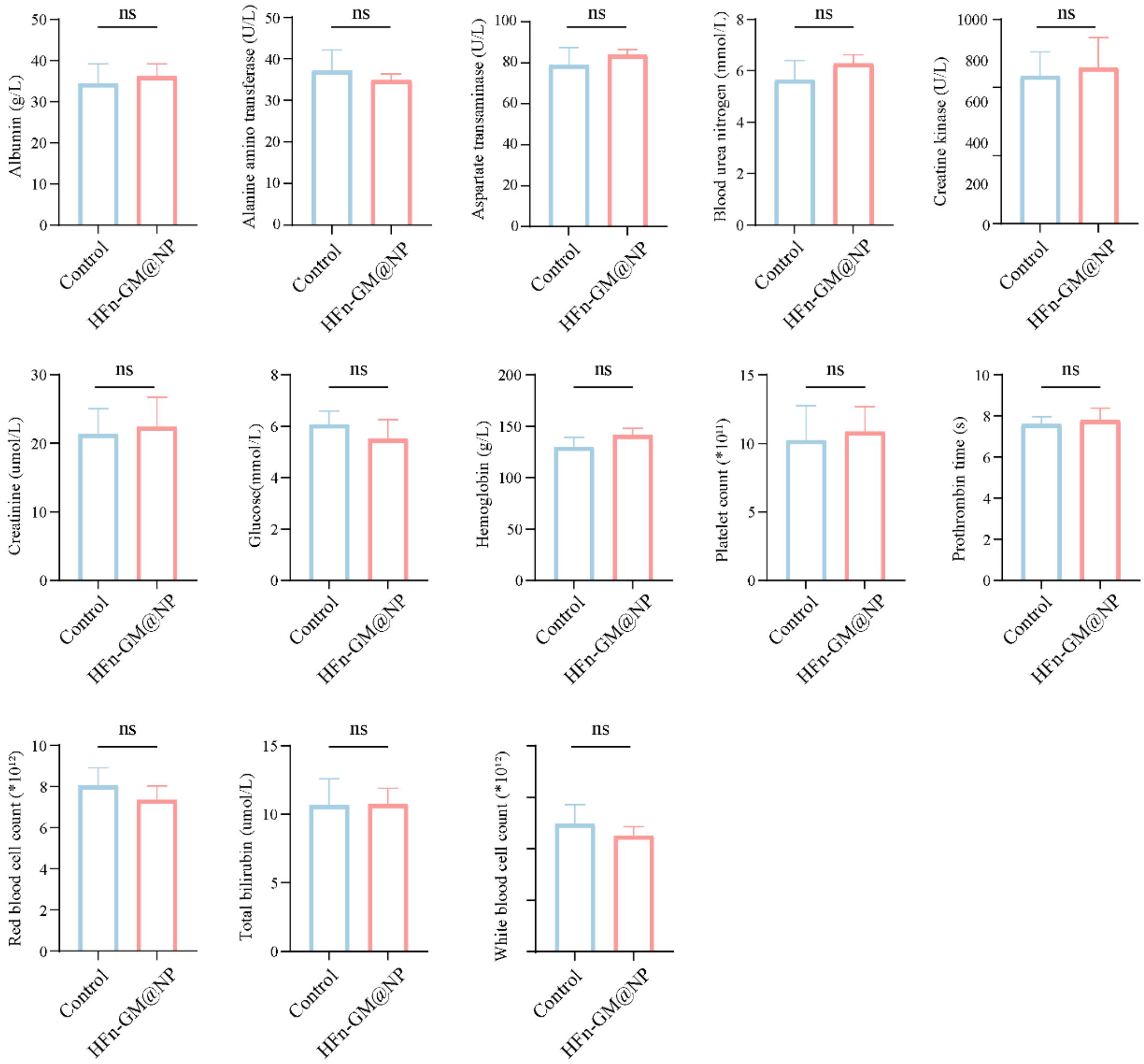

Figure S10. Hematological parameter analysis for the assessment of HFn-GM@NP biosafety in mice (n=3).

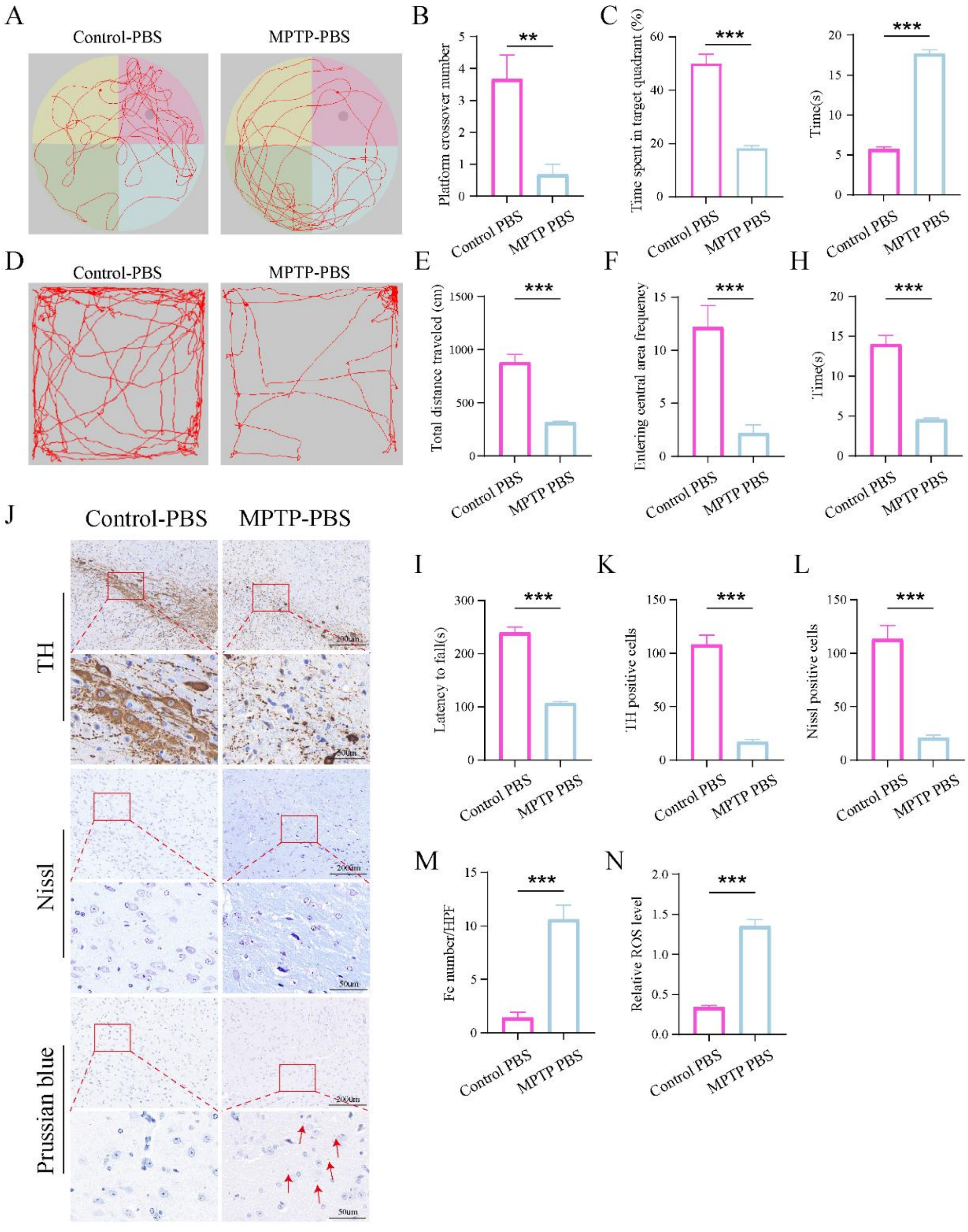

Figure S11. Comparison of motor and neuropathological features between non-MPTP healthy control mice and MPTP/PBS mice. (A) Representative swimming trajectories of mice in the Morris water maze test; (B) Statistical comparison of the number of platform crossings (n=6);(C) The relative percentage of time spent in the target quadrant (n=6); (D) Representative locomotor trajectories of mice from different treatment groups in the open field test; (E) Quantification of the total distance traveled by mice (n=6); (F) Statistical analysis of the number of entries into the central zone

(n=6); (G) Statistical analysis of the time required for mice to descend the pole (n=6); (H) Comparison of hanging time among mice (n=6); (I) Comparison of rotarod latency among mice (n=6). (J) Representative images of TH staining, Nissl staining, and Prussian blue iron staining of the substantia nigra in mouse brain. (K-M) Quantitative analysis of TH staining, Nissl staining, and Prussian blue iron staining (n=6). (N) ELISA based quantification of ROS levels in the mouse substantia nigra (n=6). The MPTP-PBS group corresponds to the PBS group in Figures 5 and 6, and shares representative images. *P < 0.05, **P < 0.01, and ***P < 0.001

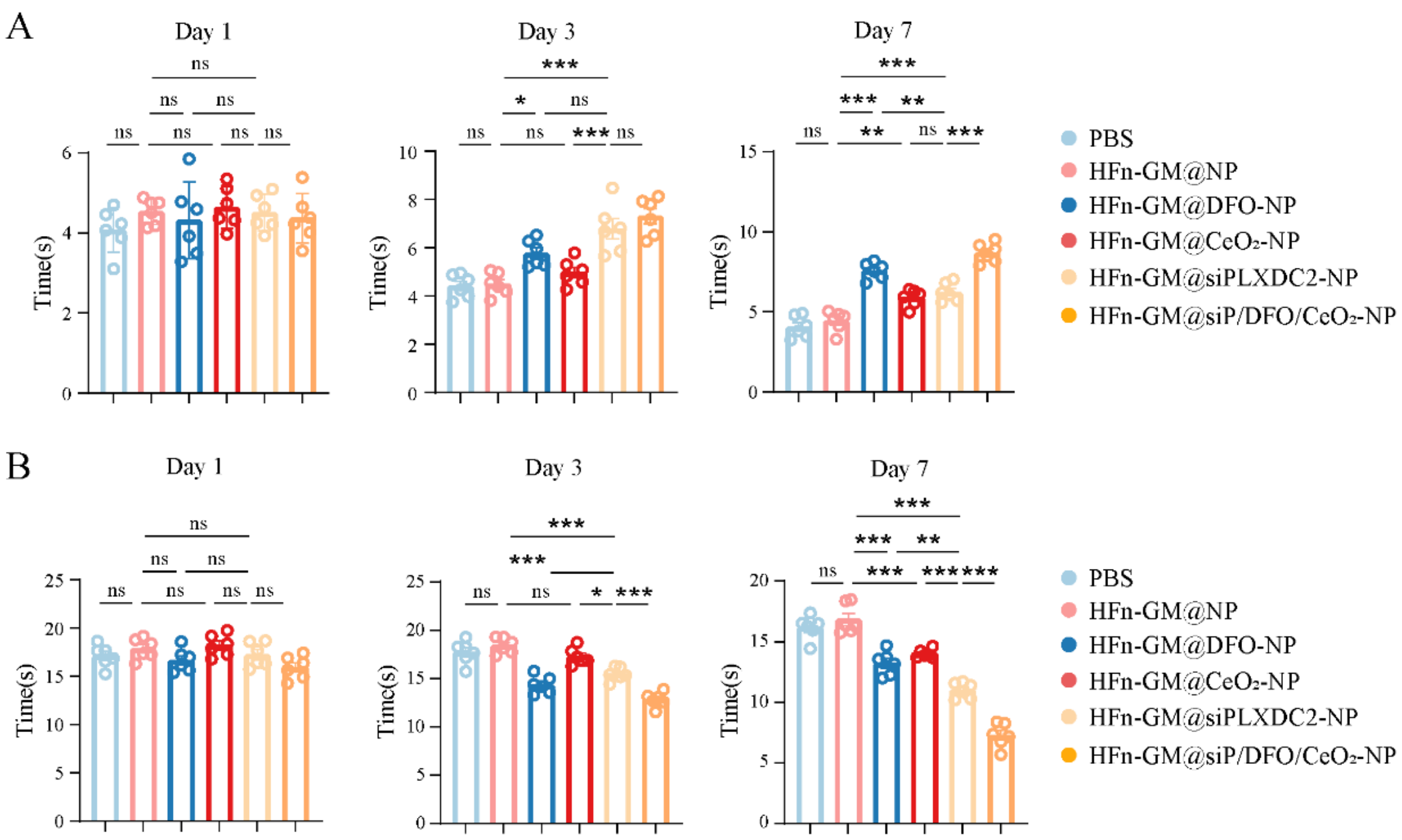

Figure S12. Evaluation of motor function in PD mice receiving different treatments. (A) Traction test assessing hanging time on days 1, 3, and 7 after treatment (n=6). **(B)** Pole test assessing the time required to descend the pole on days 1, 3, and 7 after treatment (n=6). *P < 0.05, **P < 0.01, and ***P < 0.001

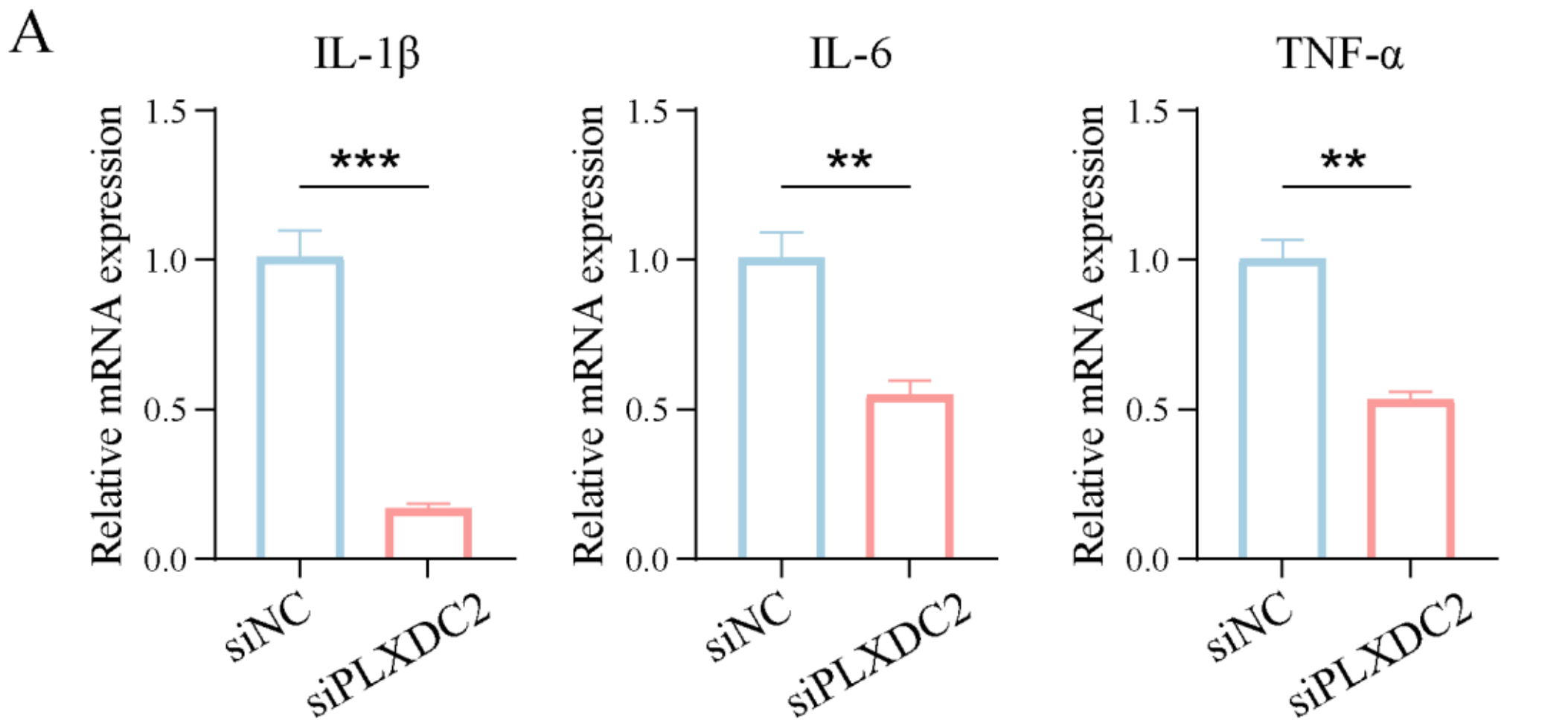

Figure S13. Statistical analysis of BV2 cells. (A) qPCR analysis of neuroinflammatory cytokine expression in BV2 cells (n=3). (B) Quantitative analysis of senescence-associated protein expression by Western blot detection (n=3). *$P < 0.05$, **$P < 0.01$, and ***$P < 0.001$

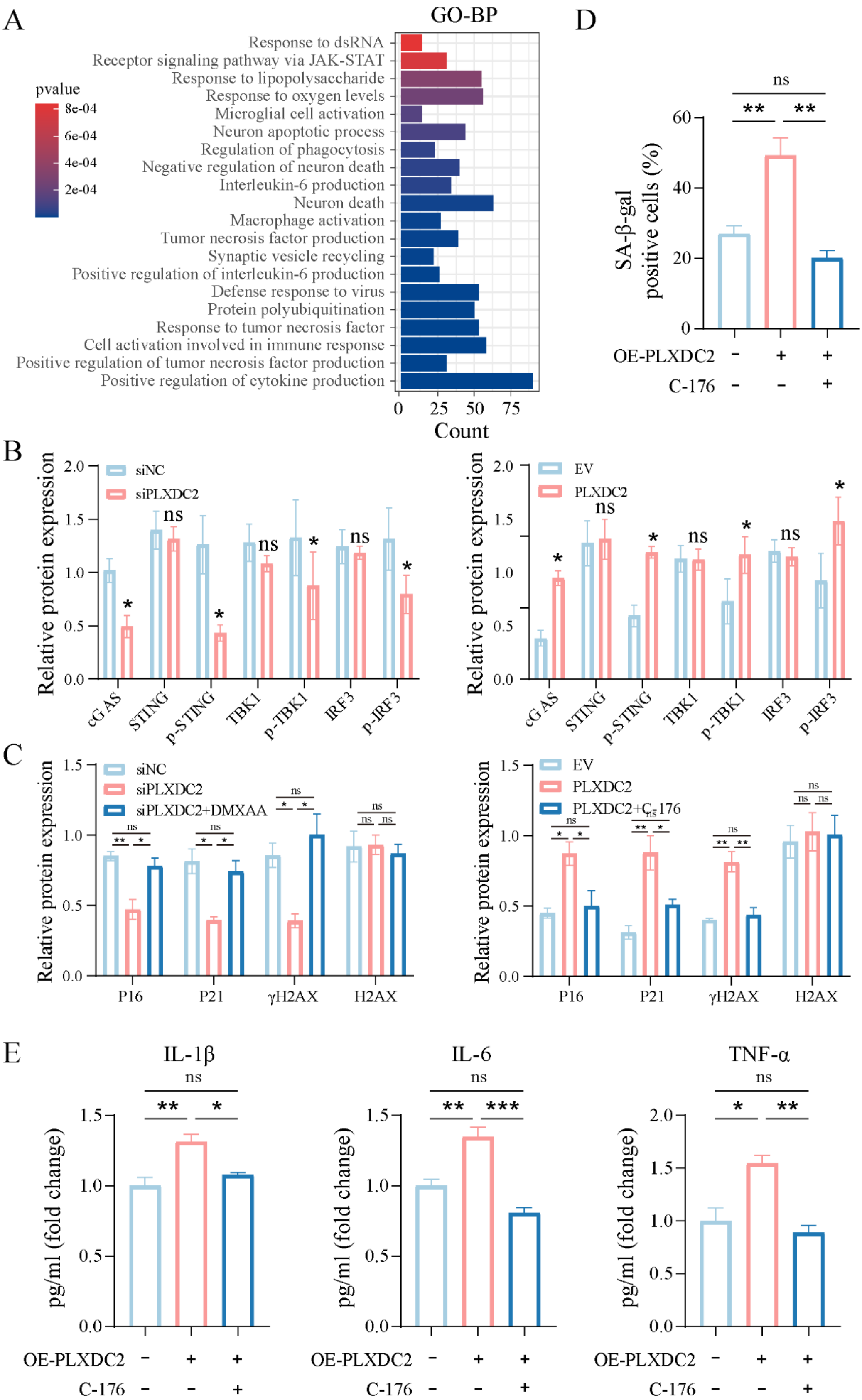
A
GO-BP
pvalue
8e-04
6e-04
4e-04
2e-04
Response to dsRNA
Receptor signaling pathway via JAK-STAT
Response to lipopolysaccharide
Response to oxygen levels
Microglial cell activation
Neuron apoptotic process
Regulation of phagocytosis
Negative regulation of neuron death
Interleukin-6 production
Neuron death
Macrophage activation
Tumor necrosis factor production
Synaptic vesicle recycling
Positive regulation of interleukin-6 production
Defense response to virus
Protein polyubiquitination
Response to tumor necrosis factor
Cell activation involved in immune response
Positive regulation of tumor necrosis factor production
Positive regulation of cytokine production
0 25 50 75
Count
D
SA-β-gal positive cells (%)
OE-PLXDC2
C-176
B
Relative protein expression
siNC
siPLXDC2
EV
PLXDC2
cGAS
STING
p-STING
TBK1
p-TBK1
IRF3
p-IRF3
C
siPLXDC2+DMXAA
PLXDC2+C-176
P16
P21
γH2AX
H2AX
E
IL-1β
IL-6
TNF-α
pg/ml (fold change)

Figure S14. Role of cGAS-STING signaling in PLXDC2 mediated microglial senescence. (A) GO biological process enrichment analysis of differentially expressed genes between high and low PLXDC2 expression groups. (B) Quantitative analysis of cGAS-STING protein expression by Western blot detection (n=3). (C) Quantitative analysis of senescence-associated protein expression by Western blot detection (n=3). **(D)** Quantification of SA-β-gal positive cells (n=3). **(E)** ELISA measurement of neuroinflammatory cytokines in BV2 cell culture supernatants (n=3). *P < 0.05, **P < 0.01, and ***P < 0.001

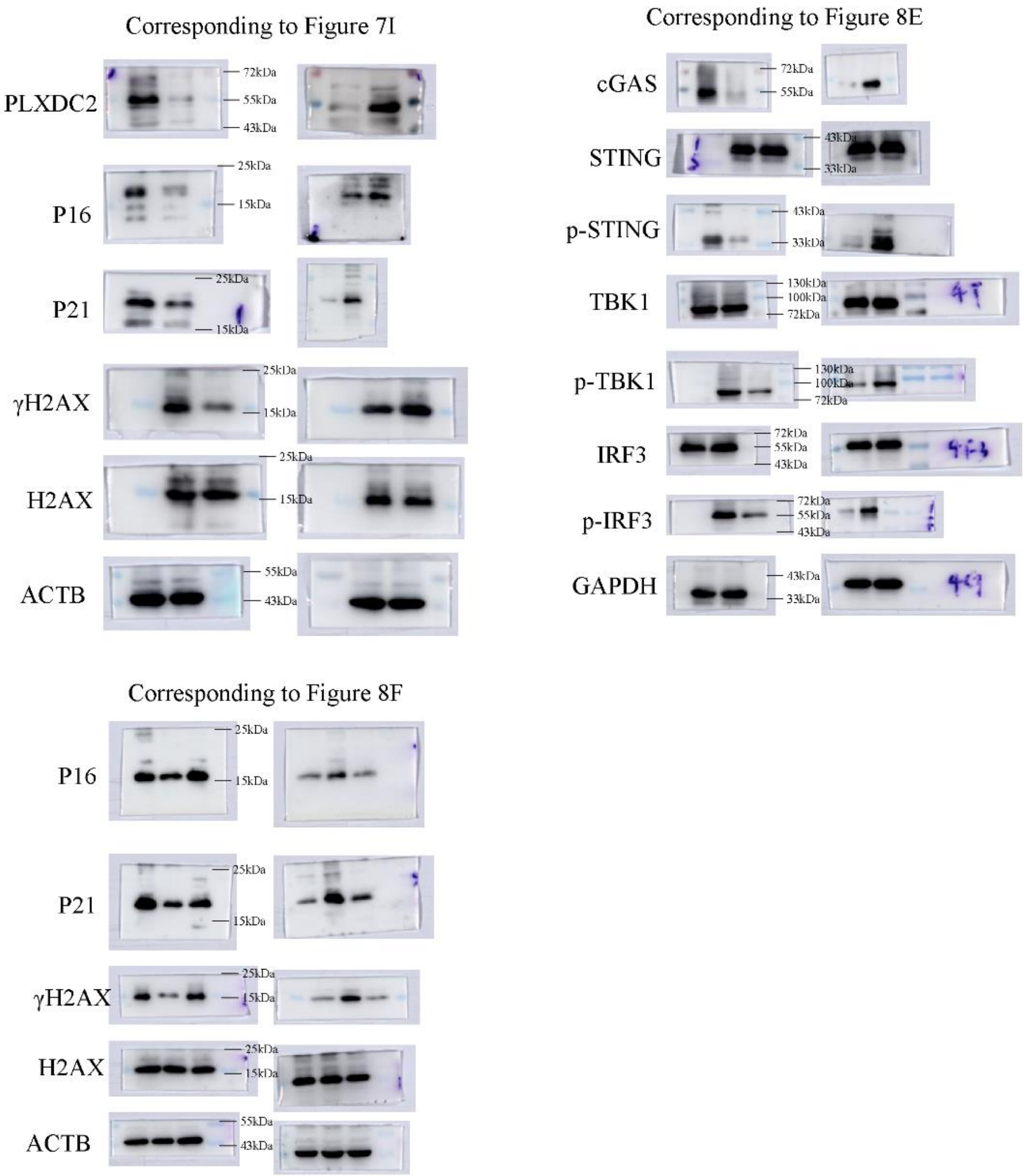

Figure S15. The original images from the Western blot analysis in this study.
